# Engineering Smart Composite Hydrogels for Wearable Disease Monitoring

**DOI:** 10.1007/s40820-023-01079-5

**Published:** 2023-04-15

**Authors:** Jianye Li, Qiongling Ding, Hao Wang, Zixuan Wu, Xuchun Gui, Chunwei Li, Ning Hu, Kai Tao, Jin Wu

**Affiliations:** 1 State Key Laboratory of Optoelectronic Materials and Technologies and the Guangdong Province Key Laboratory of Display Material and Technology, School of Electronics and Information Technology, Sun Yat-Sen University, Guangzhou, 510275 People’s Republic of China; 2 Ministry of Education Key Laboratory of Micro and Nano Systems for Aerospace, Northwestern Polytechnical University, Xi’an, 710072 People’s Republic of China; 3 Department of Chemistry, Zhejiang University, Hangzhou, 310058 People’s Republic of China; 4 ZJU-Hangzhou Global Scientific and Technological Innovation Center, Zhejiang University, Hangzhou, 311215 People’s Republic of China; 5 Department of Otolaryngology, The First Affiliated Hospital, Sun Yat-Sen University, Guangzhou, 510275 People’s Republic of China

**Keywords:** Wearable health monitoring, Smart composite hydrogel, Hydrogel engineering, Wearable sensor, Flexible and stretchable sensors

## Abstract

Growing health awareness triggers the public’s concern about health problems. People want a timely and comprehensive picture of their condition without frequent trips to the hospital for costly and cumbersome general check-ups. The wearable technique provides a continuous measurement method for health monitoring by tracking a person’s physiological data and analyzing it locally or remotely. During the health monitoring process, different kinds of sensors convert physiological signals into electrical or optical signals that can be recorded and transmitted, consequently playing a crucial role in wearable techniques. Wearable application scenarios usually require sensors to possess excellent flexibility and stretchability. Thus, designing flexible and stretchable sensors with reliable performance is the key to wearable technology. Smart composite hydrogels, which have tunable electrical properties, mechanical properties, biocompatibility, and multi-stimulus sensitivity, are one of the best sensitive materials for wearable health monitoring. This review summarizes the common synthetic and performance optimization strategies of smart composite hydrogels and focuses on the current application of smart composite hydrogels in the field of wearable health monitoring.

## Highlights

- The common performance optimization strategies of smart composite hydrogel are summarized.
- The recent advanced progress of smart composite hydrogel-based wearable sensors is systematically discussed from the aspect of health monitoring.
- The current challenges and future prospects of smart composite hydrogel-based wearable sensors are presented.

## Graphical abstract

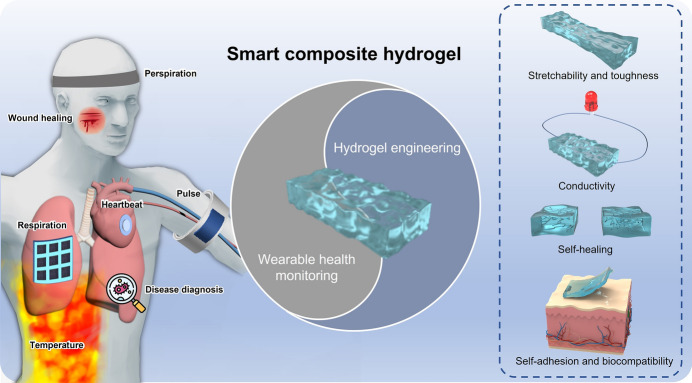

## Introduction

Nowadays, health problems are getting increasingly prominent. Various chronic diseases have become the primary threat to public health and, at the same time, show a younger trend [1, 2]. It is necessary to have a timely and comprehensive understanding of one's physical condition in the context of increasingly severe health problems. However, traditional health monitoring methods usually rely on colossal equipment and professional medical personnel, resulting in inconvenient monitoring and high costs. The emergence of wearable health monitoring technology has fixed these problems quite well [3–5]. The wearable monitoring system can record the user's physical information in real time, evaluate the user's health level and provide personalized medical advice through data analysis, which is of great significance in medical care, disease prevention and control. In the whole process of health monitoring, the recording and conversion of signals are very important, and it determines whether the information obtained by the user is reliable. Therefore, how to prepare sensors with reliable performance is a topic that researchers are very concerned about.

Currently, the internal sensors of commercial wearable health monitoring devices such as smartwatches and smart glasses are manufactured using semiconductor processes based on rigid substrates, resulting in difficulty in adapting to the surface of the human body and low wearing comfort [6, 7]. The limited health information they collect, such as body temperature, pulse and blood oxygen, renders them hard to adapt to increasingly diversified and personalized healthcare scenarios. In addition, a large Young's modulus gap exists between these rigid sensors and the human skin surface. Rigid sensors tend to detach from the body surface when a person makes large movements, which can lead to inaccurate data recording [8]. Therefore, developing a fully flexible and stretchable sensor is crucial for constructing a wearable health monitoring system.

There are many strategies for achieving fully stretchable and flexible sensors. For example, some researchers directly deposit thin sensitive materials with low Young's modulus (usually inorganic materials) on flexible substrates while improving their stretchability through geometrical design, such as infiltrated serpentine structures, wavy structures, and island bridge structures [9]. Other researchers directly use stretchable and flexible sensitive materials to fabricate wearable sensors. Stretchable and flexible sensitive materials are usually prepared by filling specific substances into an elastic matrix, such as styrene-butadiene rubber (SBR), polyurethane (PU), hydrogel and poly(dimethylsiloxane) (PDMS) [10–12]. The first method dedicates to improving stretchability through geometric structural design. Its manufacturing process is usually complicated and costly. More importantly, the stretchability of the sensor prepared by this method is very limited. Thus, researchers generally tend to use the second scheme.

Common flexible and stretchable materials can be divided into solvent-free or water-free cured elastomers, such as polyamide and polyurethane, and organic hydrogel or hydrogel swelled with a large amount of organic solvent or water inside [13–16]. Although both have good flexibility and stretchability, hydrogels comprise a three-dimensional polymer network and a large amount of water inside the network, having a structure similar to soft tissue. Thus, hydrogels can contact skin or organ surfaces for extended periods without causing tissue inflammation or ulceration and are more suitable for monitoring human signals [17]. Meanwhile, the 3D porous structure inside the hydrogel can carry a variety of sensitive factors and then be endowed with multi-stimuli responsiveness to respond to external stimuli and have high sensitivity. Compared with commercial sensors based on traditional semiconductor processes and elastomer-based sensors, the hydrogel-based sensor has attracted increasing attention in wearable health monitoring due to its good mechanical properties, biocompatibility, flexible and adjustable characteristics.

However, the properties of traditional hydrogels are minimal and cannot meet the increasingly diversified application requirements [18–21]. Researchers hope to improve the performance of hydrogels by adjusting the internal components or using specific mechanisms to build unique internal structures. These regulated hydrogels are usually doped with organic or inorganic fillers such as nanoparticles and carbon nanotubes or contain multiple cross-linked networks, thus called composite hydrogels. Up to now, research teams around the world have carried out a lot of research work in the field of smart composite hydrogel-based sensors. For instance, in 2018, Wu et al. [22], for the first time, exploited polyacrylamide (PAM)/carrageenan double network (DN) hydrogels as temperature-sensitive materials to prepare a thermistor with outstanding mechanical properties and thermal sensitivity. In the same year, they devised a solvent replacement strategy to partially replace the water in the PAM/carrageenan hydrogel with glycerol and successfully prepare an organohydrogel with long-term stable performance [23]. Due to the combined action of glycerol (Gly) and water molecules, the DN-Gly NO_2_ sensor can maintain sensitivity for up to 9 months. In 2019, Wu et al. [24] applied PAM/carrageenan DN-Gly hydrogel to construct intrinsically stretchable and self-healable humidity sensor. The organohydrogel sensor showed high sensitivity and a wide detection range to humidity changes, producing an apparent response to relative humidity variation in the range of 4–90% relative humidity (RH).

The smart composite hydrogel-based sensors in various reports have different monitoring targets, use different sensing principles and fabrication processes, and are applied to diverse scenarios. According to the detection target, smart composite hydrogel-based sensors can be classified as gas, humidity, temperature, strain, pressure, pH sensors, and biosensors [25–34]. Based on sensing principles, smart composite hydrogel-based sensors can be divided into electrochemical sensors, colorimetric sensors, resistive sensors, capacitive sensors, etc. [35–39]. Smart composite hydrogel-based sensors with different monitoring targets, sensing principles, and internal structures adopt different manufacturing techniques. Technologies such as self-assembly, screen printing, 3D printing, electrospinning, and micromachining process have been widely used in the preparation of hydrogel-based sensors [40–44]. Besides, the highly tunable nature of composite hydrogels enables them to be used in various complex scenarios such as human–computer interaction, health monitoring, and environmental detection based on different applications [45–50].

As one of the crucial wearable application scenarios, the wearable health monitoring application of hydrogel materials has been extensively discussed in many reviews before. Fu et al. [51] reviewed recent advances in the biomedical applications of cellulose-based hydrogels from drug delivery, tissue engineering, wound dressings, bioimaging, and wearable sensors. The discussion of health monitoring mainly focuses on detecting human motions, such as a heaving chest during breathing or blood vessels pulsating. Deng et al. [52] classified and discussed the stimulus types that stimulus-responsive conductive hydrogels respond to, including temperature, pH value, near-infrared (NIR) light, magnetic field, electric field, and a variety of stimuli, and proved the feasibility of stimulus-responsive conductive hydrogels for human motion/health detection and electronic skin, on–off switching of electronic devices, actuators, controlled drug release, antibacterial wound healing and skin repair, photothermal therapy, tissue engineering and cell delivery. This review discusses health monitoring as one of the aspects and only focuses on health monitoring applications related to subtle motions of the human body. Wang et al. [53] reviewed various hydrogel-based composites but merely summarized recent progress in potential diagnostics for diabetes, cancer, and cardiovascular diseases. Most existing reviews related to hydrogel wearable health monitoring are limited to one aspect or based on a specific type of hydrogel. Therefore, systematic summaries of smart composite hydrogel-based wearable health monitoring applications are needed.

**Fig. 1:**
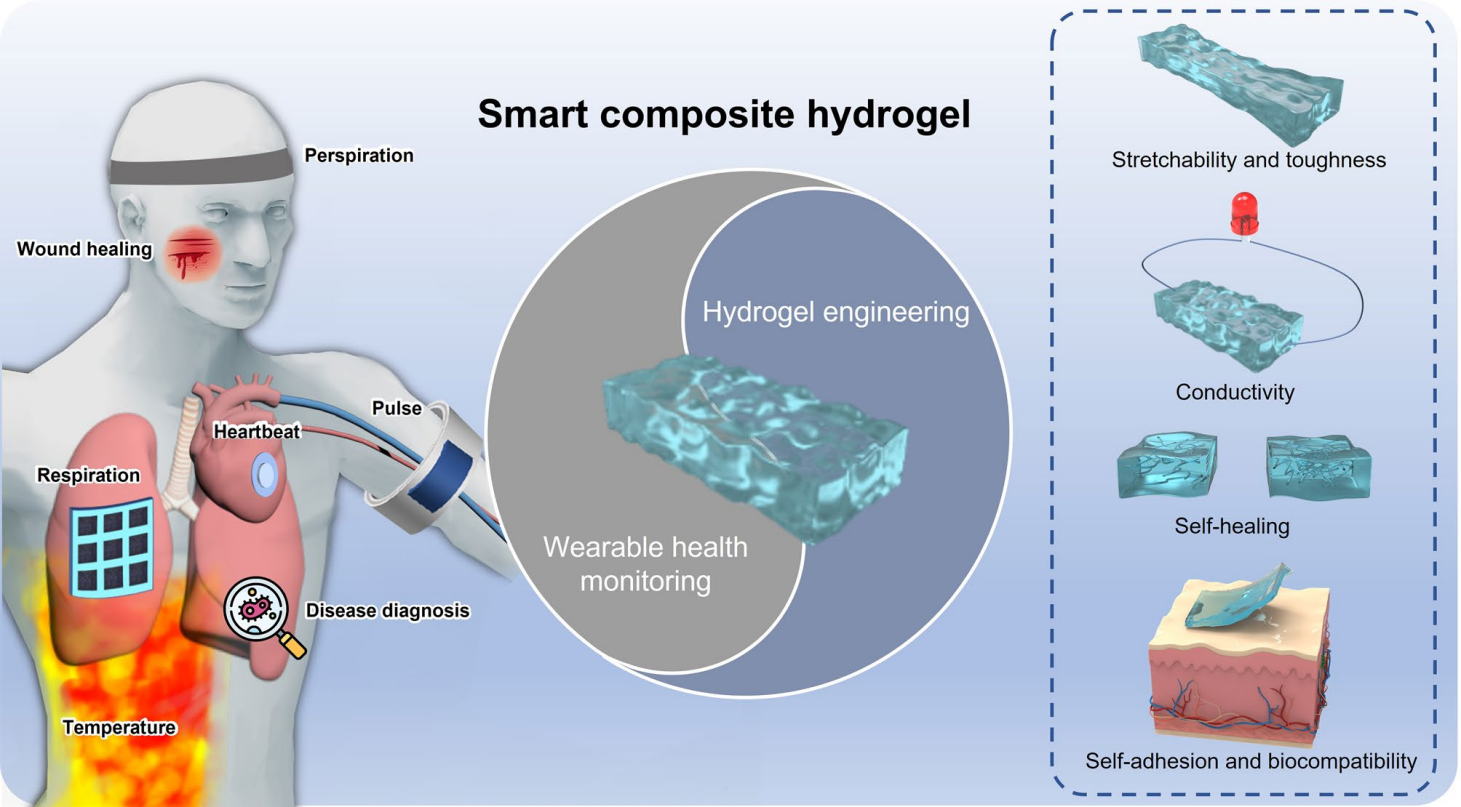
Engineering smart composite hydrogels for wearable health monitoring

In this review, we first overview the common synthesis mechanisms of composite hydrogels, including physical cross-linking polymerization, chemical cross-linking polymerization and chemical-physical mixed cross-linking polymerization. Then we put forward the concept of hydrogel engineering and further introduce the strategies to improve the properties of composite hydrogels. Additionally, we summarize recent advanced progress of smart composite hydrogel-based wearable sensors from the aspect of health monitoring (Fig. 1). The last part is a review of the entire essay and an outlook on the future development of smart composite hydrogel-based sensors.

## Design Strategies of Composite Hydrogels

### Synthesis Mechanisms

Organic monomers polymerize in water to form long molecular chains. These long molecular chains cross-link, entangle each other, and encapsulate a large amount of water in the formed three-dimensional structure, thereby preparing a hydrogel with a certain shape. The polymer network inside the hydrogel acts as a skeleton and mainly plays a supporting role. These three-dimensional polymer networks are constructed via cross-linking of polymer chains. Cross-linking mechanisms can significantly affect the mechanical properties, conductivity and many other properties of hydrogels. According to the different action forms during network bonding, the hydrogel synthesis mechanism can be divided into physical and chemical cross-linking [54]. The hydrogels prepared by these two mechanisms are physically cross-linked hydrogels and chemically cross-linked hydrogels.

Physically cross-linked hydrogels are prepared through physical interactions such as ionic bonds, hydrogen bonds, molecular self-assembly, and chain entanglement [55–57]. Inside physical hydrogels, the internal dynamic non-covalent interactions are reversible, which enables the polymer chain network to be unconnected or reconnected under certain conditions [58]. For example, some thermally responsive polymers, such as gelatin, polyacrylic acid and poly(*N*-isopropylacrylamide) (PNIPAAM), undergo physical entanglement as the temperature changes. This change is usually caused by a change in their solubility and the formation of packed polymer backbones that are physically rigid [59, 60]. An increase or decrease in temperature results in thermal gelation, where the transition temperature is defined as lower critical solution temperature (LCST) and higher critical solution temperature (UCST), respectively [61, 62]. For macromolecules with LCST properties, the precursor solution gels when the temperature is higher than LCST. Barbara L. Ekerdt et al. [63] proposed a 3D biomaterial composed of hyaluronic acid and PNIPAAM with an LCST of 37 °C. The hyaluronic acid-poly(N-isopropylacrylamide) (HA-PNIPAAM) hydrogel with thermoresponsive properties mixes with cells at low temperatures, encapsulates cells when the temperature is higher than LCST (37 °C), and recovers cells upon cooling and reliquefaction. HA-PNIPAAM, as a polymer-synthesized media free of animal components, can effectively prevent the contamination of hPSC cells during the culture process. Unlike macromolecules with LCST properties, phase transition does not occur in macromolecules with UCST properties until the temperature drops below UCST. Nonionic polymers containing primary amide groups, such as poly(N-acryloyl glycinamide) (poly(NAGA)), can exhibit UCST behavior in aqueous solutions due to thermoreversible hydrogen bonding. However, the UCST behavior of poly(NAGA) had not been noticed in the long past because of the inadvertently introduced ionic groups. In 2011, Seema et al. [64] successfully demonstrated that the UCST behavior of poly(NAGA) in water was suppressed by trace ionic groups introduced unintentionally by monomer impurities prior to polymerization, by polymer hydrolysis, by the use of ionic radical initiators or ionic chain transfer agents. This work establishes a synthetic and analytical basis for developing poly(NAGA) hydrogels with intrinsic UCST.

**Fig. 2:**
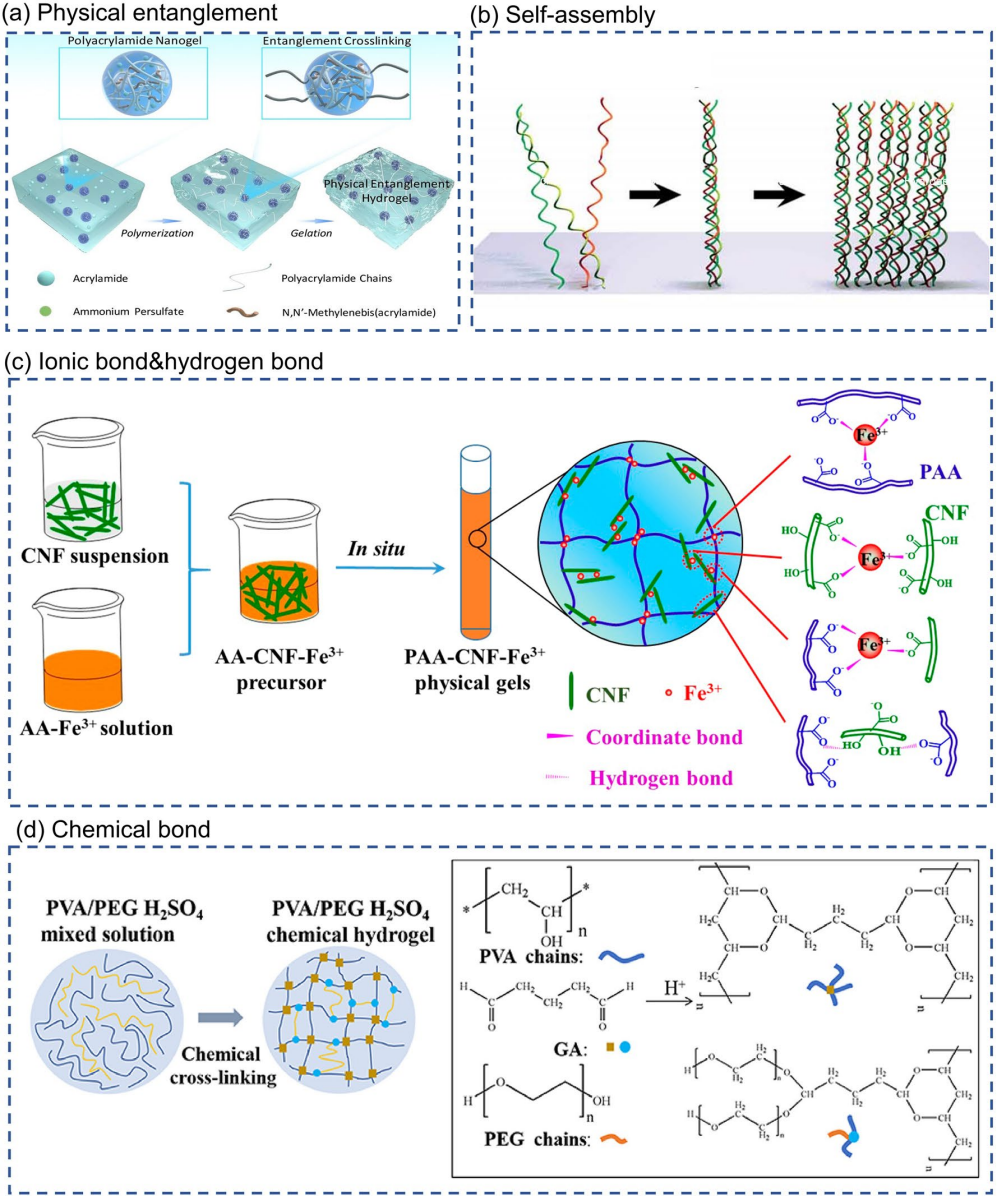
Synthesis mechanisms of hydrogel. **a** Physical entanglement. Schematical illustration of the formation of physical entanglement hydrogels (PEH). Polyacrylamide nanogels absorbed acrylamide monomer and initiator which undergo in situ polymerization to form “entanglement cluster” to gel the system [65]. Reproduced with permission. Copyright 2010, Royal Society of Chemistry. **b** Self-assembly [21]. Reproduced with permission. Copyright 2017, American Chemical Society. **c** Ionic bond and hydrogen bond. Schematic of one-pot preparation of PAA-CNF-Fe^3+^ physical gels and illustrations of hydrogen bonds and dual coordinate bonds [66]. Reproduced with permission. Copyright 2017, American Chemical Society. **d** Chemical bond. The chemical cross-linking process of PVA/PEG H_2_SO_4_ chemical hydrogel [70]. Reproduced with permission. Copyright 2020, Elsevier

Physical entanglement is an extremely common interaction in hydrogels. However, physical entanglement is considered to be too weak to build robust hydrogel networks solely by itself. Herein, Cui et al. [65] devised a cluster strategy for preparing a new class of hydrogels cross-linked through physical entanglement interactions of clusters. Schematical illustration of the formation of physical entanglement hydrogels is shown in Fig. 2a. Dense entanglements called "clusters" are created in the highly cross-linked nanogels to provide sufficiently strong interactions. In addition to the physical entanglement mentioned above, hydrogen and ionic bonds are common factors that promote the formation of cross-linked structures of molecular chains in physical gels. For example, Shao et al. [66] prepare a fully physically cross-linked poly(acrylic acid)-cellulose nanofibrils-Fe^3+^ (PAA-CNF-Fe^3+^) hydrogel by a simple one-pot strategy. Figure 2c is schematic of one-pot preparation of PAA-CNF-Fe^3+^ physical gels and illustrations of hydrogen bonds and dual coordinate bonds. Inside the PAA-CNF-Fe^3+^ hydrogel, the iron ions (Fe^3+^) and 2, 2, 6, 6-tetramethylpiperidine-1-oxyl radical (TEMPO) oxidized CNFs act as cross-linking agents, and PAA act as polymer chains. CNFs and PAA chains form the first cross-linked network induced by hydrogen bonding. The dual coordination of Fe^3+^ with the carboxyl groups in PAA and carboxylated CNFs results in the formation of a second cross-linked network. The strategy of synergistic effect between multiple non-covalent bonds endows the PAA-CNF-Fe^3+^ hydrogel with self-healing properties (self-healing rate > 90%) and, at the same time, dramatically improves the hydrogel's mechanical properties.

Non-covalent molecular self-assembly is also a common strategy, especially for protein-based hydrogels (Fig. 2b). A notable example is the hierarchical self-assembly of collagen. First, the regular arrangement of specific amino acids, such as proline or hydroxyproline, in the peptide chain promotes the formation of a triple helix structure. Then, these triple helices entangled each other to form a nanofiber structure, forming collagen hydrogels via continuing self-assembly. Inspired by the hierarchical self-assembly of natural collagen, O'Leary proposed the design and synthesis of a self-assembling peptide, which formed high-quality hydrogels by replicating the multi-hierarchical self-assembly steps of collagen [67].

The polymer chains inside the chemical hydrogel are cross-linked with each other through covalent bonds. Compared with physical cross-linking, chemical cross-linking is a strong interaction that makes chemically cross-linked hydrogels solid and durable [68, 69]. Guo et al. [70] prepared a hydrogel electrolyte by chemically cross-linking reaction of polyvinyl alcohol (PVA) and polyethylene glycol (PEG) with glutaraldehyde (GA). The synthesis process of PVA-PEG hydrogel is shown in Fig. 2d. The hydroxyl group (-OH) of PVA and PEG reacts with the aldehyde group (-CHO) of GA in sulfuric acid solution to generate acetal or hemiacetal, resulting in cross-linking of polymer chains and the formation of a 3D network. GA acts like a cross-linking agent in this process. Furthermore, owing to acetal bonding in the cross-linking structure of PVA and PEG molecular, PVA-PEG hydrogels have better mechanical properties than pure PVA hydrogels. Thus, PVA-PEG hydrogels can withstand stretching, twisting and large deformations in bending.

In another work, Min Hee Kim and Won Ho Park investigated the differences in structure and properties of physically cross-linked hydrogels and chemically cross-linked hydrogels [71]. They prepared physically cross-linked silk fibroin hydrogel (SF P-gel) and chemically cross-linked silk fibroin hydrogel (SF C-gel) according to the following procedures. First, they dissolved Degummed B. mori silk fiber in a ternary solvent system consisting of calcium chloride, ethanol and water. Then, a high-concentration SF solution was obtained through a series of dialysis and centrifugation operations. Finally, the SF solution was placed at 37 °C for the gelation of SF P-gel. For the preparation of SF C-hydrogel, SF solutions were poured into a petri dish and irradiated with γ-rays from a Co-60 source. Finally, SF C-gel was formed via gamma-ray (γ-ray) irradiation-induced chemical cross-linking reactions of SF. To study the difference between SF P-gel and SF C-gel, the research team conducted compressive stress tests on SF P-gel and SF C-gel. Although the maximum compressive strength of SF C-gel was lower than that of SF P-gel in the first cycle, SF C-gel has better compressive recovery due to its low crystallinity and intermolecular cross-linking reaction. In general, the polymer chains inside chemical hydrogels interact through chemical covalent bonds. Their bonds are usually permanent and more robust. At the same time, the polymer chains inside physical hydrogels often rely on physical interactions such as ionic bonds, hydrogen bonds, and hydrophobic interactions to form a transient connection, which is not permanent and will undergo reversible changes under certain conditions. Chemical cross-linking can obtain hydrogels with better mechanical properties and stability. Nevertheless, due to toxic cross-linking agents involved in the reaction process and reactions detrimental to biological activity, chemically cross-linked hydrogels are not suitable to apply to organisms, especially in biomedical applications and wearable electronics [72]. The different synthesis mechanisms of hydrogels are summarized in Table 1.

**Table 1:** Comparison of different synthesis mechanisms

Cross-linking mechanism	Interaction force	Feature
Physical cross-linking	Ionic bond Hydrogen bond Self-assembly Physical entanglement	Reversible network Weak interaction Usually non-toxic
Chemical cross-linking	Chemical bond	Irreversible network Robust and permanent bond Better mechanical properties and stability Using toxic cross-linking agents

Along with the expansion of the application field of hydrogels, the properties of hydrogels prepared by a single cross-linking mechanism are minimal and, thus, difficult to adapt to complex and diverse application scenarios. Therefore, people tend to use multiple cross-linking mechanisms to prepare hydrogel materials with diverse properties. The free combination of various physical or chemical cross-linking mechanisms makes hydrogels more flexible and tunable, significantly expanding the application boundaries of hydrogels [73, 74]. Hu et al. [75] prepared polyacrylamide/C-dot (PAM/C-dot) hydrogel with extraordinary mechanical, recoverable, and swelling properties by incorporating carbon nanodots (C-dots) as physical cross-linking agents and lubricants into low-chemically cross-linked PAM networks. When the hydrogel is stretched, the strong and low cross-linked chemical network deforms, leading to the movement of PAM chains, and the unzipping of the intensive physical cross-linked network relieves some of the stress. Furthermore, the stretched chemical network returns to its original state when the stress is removed, while the unzipping physical cross-links can recombine. The unique internal structural changes of the hydrogel in stretched and relaxed states endow it with excellent mechanical and recovery properties. Zhang et al. [76] designed and fabricated a novel physicochemical double-crosslinked network (PCDC) hydrogel. The physically cross-linked network inside the PCDC hydrogel can be reversibly reconstructed, giving the hydrogel good tensile and self-healing properties. For chemically cross-linked network, the strength of the 3D network tends to be closely related to the cross-link density. Network strength will increase with cross-link density. The surface of the chemical cross-linking agent isocyanoethyl methacrylate (SiPU) nanoparticles contains multiple double bonds. A small amount of addition can significantly improve the cross-linking density and the strength of PCDC hydrogels. More importantly, SiPU nanoparticles are soft and easily deformable. As a chemical cross-linking agent in PCDC hydrogels, they can deform under stress and recover after stress removal, which may increase the resilience of the hydrogel. It can be seen that the synergistic effect of the physicochemical double cross-linking mechanism endows the PCDC hydrogel with excellent stretchability, strength and resilience.

### Hydrogel Engineering

The application of conventional hydrogels is very limited due to their lacking characteristics and performance deficiencies in some aspects [77]. Researchers somehow expect to improve the properties of hydrogels or impart new properties to them. Hydrogel engineering is a means to control the properties of hydrogels by adjusting the internal components of hydrogels or building unique internal structures. After performance tuning via hydrogel engineering, hydrogels exhibit excellent physicochemical properties, such as substantially enhanced mechanical properties, electrical conductivity, self-healing, stimuli-responsiveness, self-adhesion, and the possibility to undergo dynamic modulation. The hydrogels regulated by hydrogel engineering are usually doped with organic or inorganic fillers such as nanoparticles and CNTs or contain multiple cross-linked networks, thus called composite hydrogels. The following section will discuss common strategies for tuning the properties of composite hydrogels. The properties of composite hydrogels mentioned are summarized in Table 2 at the end of this section.

**Table 2:** Summary of performances of diverse composite hydrogels

Composite hydrogel	Stretchability and toughness	Conductivity	Self-adhesion	Self-healing	Biocompatibility
F127AZO@β-CD hydrogel [82]	Fracture toughness: 2.68 ± 0.69 MJ m^−3^ Tensile strength: 475 kPa Fracture strain: 2100%	–	–	–	–
PAC hydrogel [85]	Tensile strength: 385 kPa Fracture strain: 1700% Elastic modulus: 49.8 kPa	–	–	–	Yes
agar/PAM-CNFs hydrogel [86]	Fracture toughness: 1792 kJ m^−3^ Fracture strain: 1077%	–	–	–	–
NIPA–AAcNa–HPR-C hydrogel [87]	Tensile strength: 40.9 kPa Fracture strain: 912% Elastic modulus: 43.2 kPa	–	–	–	–
PEDOT: PSS-PAM hydrogel [92]	Fracture strain: 360% Elastic modulus: 80 kPa	0.01 S cm^−1^	–	–	–
PGA hydrogel [93]	Tensile strength: 94 kPa Fracture strain: 472%	0.335 S m^−1^	–	–	–
PEDOT: PSS/PVA hydrogel [94]	Fracture strain: 150% Elastic modulus: 460 kPa	10 S cm^−1^	–	–	Yes
PAA/DCh-PPy/FeCl3 hydrogel [103]	Fracture strain: 1500%	40 S cm^−1^	–	Mechanical healing: 100% in 2 min Electrical healing: 96% in 1 min	–
graphene/PAA hydrogel [104]	–	1.56 $\times$ 10^−5^ S m^−1^	–	–	–
PAM/CNF/CNT hydrogel [105]	Tensile strength: 0.32 $\pm$ 0.06 MPa Fracture strain: 140%	8.5 $\times$ 10^−4^ S cm^−1^	–	–	–
SA-Zn hydrogel [107]	Tensile strength: 0.21 MPa Fracture strain: 4200%	–	–	–	–
PSS-MUI/gelatin/Fe_3_ + hydrogel [108]	Tensile strength: 37 kPa Fracture strain: 780% Elastic modulus: 36 kPa	10.3 S cm^−1^	40.9 $\pm$ 1.5 kPa (between hydrogels and polyurethane)	Mechanical healing: 97% in 2 h Electrical healing: full-recovery	–
p(APMA-co-THMA)/Dex-CHO hydrogel [118]	Tensile strength: 70.5 kPa Fracture strain: 257% Fracture toughness: 917 J m^−2^	–	422 J m^−2^ (between hydrogels and glass)	–	Yes
OPPC/CNT hydrogel [119]	Tensile strength: 0.186 MPa Fracture strain: 164%	109 ${\Omega }$	0.02 MPa (between hydrogels and porcine skin)	Mechanical healing: 97–103% in 10 s	Yes
Surface-engineered hydrogel inspired by clingfish [120]	–	–	25 kPa and 50 J m^−2^ (between hydrogels and polyampholyte)	–	–
PDA-CNT incorporated GW-hydrogel [121]	Fracture strain: 700% Fracture toughness: 2300 J m^−2^	8.2 S m^−1^	60 kPa (between hydrogels and porcine skins)	–	–
$\alpha$ CD–nBu and βCD–Ad hydrogel [125]	–	–	–	$\alpha$ CD–nBu: 74% in 24 h βCD–Ad: 99% in 24 h	–
HG1G2 hydrogels [127]	–	–	–	self-healing into a whole plate in 48 h	–
Ngel-Odex-ADH hydrogel [128]	–	–	–	–	Yes
chitosan-alkali lignin hydrogel [135]	–	–	–	–	Yes

#### Stretchability and Toughness

Mechanical properties are one of the most concerning properties of hydrogel materials. Applications such as human–computer interaction, soft robotics, and wearable devices have spurred interest in developing tough and highly stretchable hydrogels which can accommodate human joint motion and conform to complex surfaces of various substrates. Common indicators to evaluate the mechanical properties of hydrogel materials include breaking elongation, tensile strength, elasticity modulus, and fracture toughness.

**Breaking Elongation (Facture Strain).** Breaking elongation is the ratio of the max change in length when breaking to the initial length, representing the maximum deformation capacity of the material.

1 $$\begin{array}{*{20}c} {\varepsilon_{\max } = \frac{{\Delta l_{\max } }}{{l_{0} }} \times 100\% } \\ \end{array}$$

**Tensile Strength.** Tensile strength refers to the ratio of the force to the cross-sectional area of the material when it breaks and is used to characterize the maximum engineering stress the material can withstand.

2 $$\begin{array}{*{20}c} {\sigma_{\max } = \frac{{F_{\max } }}{S}} \\ \end{array}$$

**Elastic Modulus.** Elastic modulus reflects the material's resistance to elastic deformation. The larger the elastic modulus, the smaller the elastic deformation produced under the same stress state. The elastic modulus is usually defined as the ratio of stress to strain in the same direction.

3 $$\begin{array}{*{20}c} {E = \frac{\sigma }{\varepsilon }} \\ \end{array}$$

**Fracture Toughness. **Fracture toughness is the area under the stress–strain curve, which means the work absorbed by the unit volume of material before stretching to fracture. It integratively reflects the strength and the ductility of the material.

4 $$\begin{array}{*{20}c} {T = \mathop {\lim }\limits_{\varepsilon \to \infty } \mathop \int \limits_{0}^{\varepsilon } \sigma \left( \varepsilon \right){\text{d}}\varepsilon } \\ \end{array}$$

The mechanical properties of hydrogels mainly depend on the internal cross-linked network structure. The three-dimensional polymer network of super stretchable and tough hydrogels can provide good energy dissipation channels so that the hydrogels will not break readily when subjected to large deformations or enormous tensile stresses [78, 79]. It can be seen that the key to designing ultra-stretchable and tough hydrogels lies in how to construct a suitable cross-linked network structure for stress release. Many researchers have studied the fracture behavior of traditional hydrogels under ultimate stretching, and it is widely believed that excessive cross-linking is one of the critical reasons for network fracture [80]. When the concentration of cross-linking agent is high, structural inhomogeneity appears inside the hydrogel, manifested as a heterogeneous aggregation of cross-linking points and random distribution of molecular chain lengths between cross-linking points. When the hydrogel is stretched, the shortest chains tend to tighten first (similar to Cannikin Law) so that most of the stress is concentrated in these weak zones, eventually leading to premature rupture of the hydrogel.

**Fig. 3:**
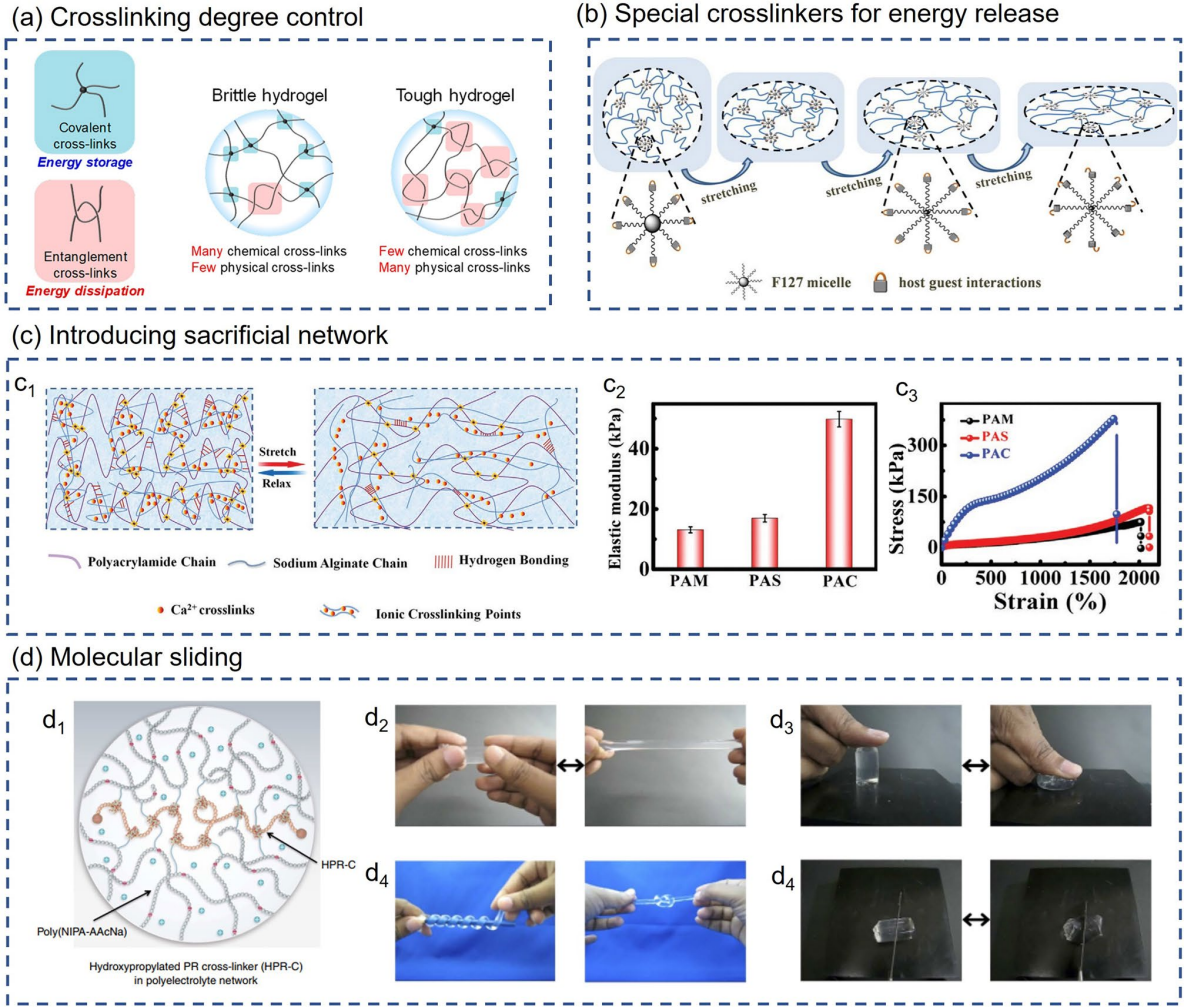
Mechanisms of regulating mechanical properties of hydrogels. **a** Cross-linking degree control. (Left) Covalent and entanglement cross-links inside the hydrogel play the roles of energy storage and dissipation. (Right) Differences in cross-linking structures of brittle and tough hydrogels [81]. Reproduced with permission. Copyright 2021, The Author(s), Springer Nature. **b** Special cross-linkers for energy release [82]. The energy release mode of F127AZO@β-CD multi-functional cross-linkers during stretching process. Reproduced with permission. Copyright 2018, American Chemical Society. **c** Introducing sacrificial network [85]. **c**_**1**_ Schematic diagram of the variation of the double network in the PAC hydrogel under stretching and releasing. **c**_**2**_ Comparison of the tensile stress–strain curves of PAM, PAS and PAC hydrogels. **c**_**3**_ Elastic modulus of three kinds of hydrogels. Reproduced with permission. Copyright 2019, John Wiley and Sons. **d** Molecular sliding [87]. **d**_**1**_ Schematic of swollen slide-ring polyrotaxane cross-linkers (HPR-C) in the NIPA–AAcNa–HPR-C hydrogel. **d**_**2**_ Elongated state of the NIPA–AAcNa–HPR-C hydrogel. **d**_**3**_ Compressed state of the NIPA–AAcNa–HPR-C hydrogel. **d**_**4**_ Coiled and knotted states of the NIPA–AAcNa–HPR-C hydrogel. **d**_**5**_ The NIPA–AAcNaHPR-C hydrogel could not be easily cut with a knife. Reproduced with permission. Copyright 2014, The Author(s), Springer Nature

Aiming at the problem of excessively high cross-linking, some researchers try to control the cross-linking degree by adjusting the content of cross-linking agents, thereby improving the mechanical properties of hydrogels. Norioka et al. [81] reduced cross-linking points and formed more polymer chain entanglement to achieve better energy dissipation by increasing the monomer concentration and, at the same time, reducing the cross-linking agent content. It can be seen from Fig. 3a that the covalent and entanglement cross-links inside the hydrogel play the roles of energy storage and dissipation, respectively. When the hydrogel is stretched, a large amount of energy is stored in the chemical covalent cross-linking point. Without sufficient physical cross-links, such as polymer entanglement, to provide energy dissipation channels, the covalent bonds formed by chemical cross-links can easily break, resulting in low fracture energy and stretchability. To demonstrate the generalization ability of this strategy, the research team used acrylamide (AM) and 2-(methacryloyloxy) ethyl phosphorylcholine (MPC) as monomers to prepare PAM and PMPC hydrogels, and investigated the influence of different cross-linking agents and monomer content on the mechanical properties of these two hydrogels. The experimental result shows that the PAM hydrogel with a cross-linker content higher than 0.1 mol% will break under slight stretching, while the PAM hydrogel with a monomer concentration of 2.5 mol L^−1^ and a cross-linker content of less than 0.01 mol% can be stretched to more than ten times the length and has the highest fracture strain. Likewise, PMPC hydrogels with low cross-linker content exhibited more excellent elongation and higher fracture strain, which confirms the feasibility of improving the stretchability and toughness by reducing the cross-linking agent content. The Young’s modulus of the PAM hydrogels prepared with the cross-linker content of 5.0 mol% and the AM concentration of 1.0, 2.5 and 5.0 mol L^−1^ were 9.6, 40.0 and 196.7 kPa, respectively. When the MPC concentration was 2.5, 5 and 10 mol L^−1^, and the cross-linker content was 0.1 mol%, the Young's moduli of the prepared PMPC hydrogels were 16.6, 60.4 and 74.0 kPa, respectively. These results indicate that excessively high monomer concentration during the polymerization process can cause massive aggregation and entanglement of physical chains. These physical entanglements can effectively release energy during the stretching process, significantly improving the mechanical properties of the hydrogel.

As mentioned before, the poor mechanical properties of conventional hydrogels are mainly due to a large number of rigid chemical cross-linker nodes (like a knotted rope). For this reason, some research teams have adopted a different solution, using a special cross-linking agent to endow the cross-linked node with energy release ability. Song et al. [82] proposed a novel supramolecular cross-linking agent that can provide special energy release channels. The F127AZO@β-CD multi-functional cross-linkers (HPCs) have many vinyl groups on the shell, which can act as cross-linkers for F127AZO@β-CD-PAM hydrogel polymerization. When the hydrogel is stretched, the energy release mode of the multi-functional cross-linking agent is shown in Fig. 3b. As the hydrogel keeps being stretched, the hydrophobic core of F127AZO@β-CD HPCs decomposes to dissipate energy. Then the host–guest switch is unlocked, again providing the hydrogel with second energy dissipation. As a result, hydrogel with multiple dissipative mechanisms exhibits excellent mechanical properties (fracture toughness of 2.68 ± 0.69 MJ m^−3^, tensile strength up to 475 kPa, and uniaxial tensile strain over 2100%).

Another way to improve the mechanical properties is to introduce sacrificial bonds into the hydrogels. These sacrificial bonds will fracture under extreme tensile conditions and release much energy, thereby increasing the hydrogels’ fracture energy and maximum tensile strain [83]. A common strategy is to introduce a double network structure into the hydrogel, in which a densely cross-linked and brittle 1st network acts as a sacrificial structure, breaking under relatively low stress to complete the dissipation of energy, and a ductile 2nd network withstands stress through large extension [84]. A double network hydrogel consisting of covalently and ionically cross-linked networks is prepared by Sun et al. [85]. The polyacrylamide–calcium alginate (PAC) DN hydrogel uses dynamic SA-Ca^2+^ (sodium alginate-Ca^2+^) bonds as sacrificial bonds for energy dissipation, and its internal polyacrylamide (PAM) chains can prevent the propagation of cracks, imparting hydrogels with outstanding mechanical performances (Fig. 3c_1_). The article also compared the mechanical features of pure PAM hydrogel, PAM-SA (PAS) hydrogel and PAC hydrogel. Figure 3c_2_ shows the tensile stress–strain curves of PAM, PAS, and PAC hydrogels. It can be seen from Fig. 3c_2_ that the stress–strain curves of PAM and PAS are almost the same, which indicates that uncross-linked SA can hardly enhance the mechanical performances of the composite hydrogel. Compared with PAS hydrogel ($\approx$ 116 kPa), the tensile strength of PAC hydrogel increased by 332% (although the maximum elongation of the PAC hydrogel was slightly decreased). Figure 3c_3_ shows that PAC hydrogels have a higher elastic modulus than PAM and PAS hydrogels, indicating that SA-Ca^2+^ bonds can significantly improve the tensile strength without drastically reducing other tensile properties.

In addition, Pan et al. [86] successfully synthesized mechanically enhanced ultra-stretchable agar/polyacrylamide (agar/PAM) DN polymer hydrogels reinforced by three different carbon nanomaterials (carbon nanofibers (CNFs), graphene nanoplatelet (GNPs), and carbon nanotubes (CNTs)) through a facile one-pot methodology. The physically cross-linked agar network inside the hydrogel acts as a sacrificial bond, and the conductive CNFs/GNPs/CNTs act as dynamic cross-linking points, effectively transmitting stress during stretching. The synergistic effect of these two mechanisms greatly improves the mechanical performance of hydrogels. Besides, owing to the piezoresistive effect and higher sensitivity to strain derived from the tunneling effect between carbon nanomaterials, the strain sensor prepared with nanomaterials-incorporated agar/PAM hydrogel has great potential to be applied in human motion detection. In addition to the methods mentioned above, molecular sliding is also an effective way to improve hydrogel’s stretchability and toughness. For example, Bin Imran et al. [87] introduced slide-ring polyrotaxane cross-linkers and ionic groups into the polymer network so that the cross-linked a-cyclodextrin molecules could move along the polyethylene glycol chains, and thereby prepared surprisingly stretchable and tough hydrogels (Fig. 3d).

#### Conductivity

Hydrogels are usually composed of three-dimensional polymer networks and swollen dispersion medium [88]. Presently there are mainly two common methods for synthesizing conductive hydrogels. One is to add conductive polymers or conductive fillers into the hydrogel to form a conductive polymer network. The other is to improve the conductivity of the hydrogel medium by introducing fillers such as conductive salts, conductive particles and carbon nanotubes into the hydrogel. According to the different sources of conductivity, conductive hydrogels can be distinguished as conductive network hydrogels and conductive medium hydrogels.

Common conductive polymers include poly(3,4-ethylenedioxythiophene): polystyrene sulfonate (PEDOT: PSS), polyaniline (PANI), polypyrrole (PPy), etc. [89–91]. Composite hydrogels with good conductivity can be synthesized by self-assembly or adding specific cross-linkers to copolymerize conductive polymers and hydrophilic polymers. Lee et al. [92] prepared a stretchable conductive hydrogel by copolymerizing AM with the conductive polymer PEDOT: PSS. First, the PEDOT: PSS solute was dispersed into a mixed solution of ethylene glycol (EG) and deionized water at a volume ratio of 1:8. AM monomer, ammonium persulfate (APS), *N*, *N*′-methylene bisacrylamide (MBA), *N*, *N*, *N*′, *N*′-tetramethylethylenediamine (TEMED) were then added into the mixed solution. In this process of AM polymerization, APS and MBA are the initiator and cross-linker, respectively, and TEMED was the accelerator. The solution was poured into a glass mold and heated at 90 °C for two hours to obtain PEDOT: PSS-PAM conductive hydrogel. When the weight ratio of PEDOT: PSS is 5.49%, the maximum tensile strain of PEDOT: PSS-PAM organohydrogel is 525%, and the electrical conductivity can reach 0.01 S cm^−1^. Similarly, Hu et al. [93] successfully prepared a polyvinyl alcohol/glycerol/polyaniline gel (PGA gel) with glycerol-water as the dispersion medium and PANI as the conductive polymer. The PVA molecular chain inside the PGA gel enhances the flexibility of the gel, PANI provides a conductive path, and glycerol prevents the evaporation and condensation of water. Therefore, the PGA gel exhibits excellent mechanical capacity (fracture strain and fracture stress of 472% and 94 kPa, respectively), high electrical conductivity (0.335 S m^−1^), and antifreeze properties (− 20 °C).

In addition to using a copolymerization strategy to prepare conductive hydrogels, conductive components can also be introduced into preformed hydrogels by an in situ polymerization strategy. Inside the hydrogel, the matrix network is the backbone to support the hydrogel, while the conductive polymer network provides the conductive channels. Thus, the double-network conductive hydrogel has good electrical conductivity and stretchability, which are attributed to the intertwined double-network structure inside. Li et al. [94] prepared a dual-network conductive hydrogel using the conductive polymer PEDOT: PSS and the hydrophilic PVA. Firstly, an appropriate amount of glutaraldehyde (GA) was added to the mixed aqueous solution of PVA and PEDOT: PSS at a weight ratio of 1:1 to cross-link PVA, and then the formed hydrogel was immersed in pure acetic acid (HOAc) to agglomerate PEDOT: PSS to form a conductive network. After the gel was immersed in deionized water to replace HOAc, PEDOT: PSS/PVA double-network hydrogels were finally obtained. When the hydrogel is stretched, the fragile PEDOT: PSS network inside breaks and releases energy, while the PVA network prevents the propagation of cracks, significantly improving the tensile properties of the hydrogel. Single network hydrogels [95, 96] possess electrical conductivity comparable to PEDOT:PSS/PVA DN hydrogels, but their mechanical properties are far worse than that of DN hydrogels. Conducting polymer-based IPN (interpenetrating polymer networks) hydrogels [97–102] are as stretchable as DN hydrogels but exhibit poor electrical conductivity. Darabi et al. [103] also successfully prepared a highly stretchable conductive self-healing hydrogel (CSH) through the strategy of constructing a double network structure. The double network structure inside the conductive hydrogel is mainly composed of a chemical cross-linked network formed by the chemical polymerization of acrylic acid (AA) monomer and a physically cross-linked network formed by in-situ polymerization of polypyrrole-grafted chitosan (Dch-PPy). The prepared double-network hydrogel has a maximum tensile strain close to 2000% with the addition of 35% MBA cross-linker, and its electrical conductivity can reach 24 S cm^−1^, indicating its extremely high electrical conductivity and excellent mechanical properties.

In addition to introducing conductive polymers, improving the conductivity of hydrogel medium by adding fillers such as conductive salts, conductive particles, and carbon nanotubes is another common method to improve electrical conductivity. Alam et al. [104] prepared an electrically conductive and mechanically strong composite hydrogel by in-situ polymerizing AA and graphene sheets. The addition of graphene significantly improved the material's compressive strength and electrical conductivity. Chen et al. [105] successfully prepared polyacrylamide/cellulose nanofibers/carbon nanotube (PAM/CNF/CNT) hydrogels by using MBA as cross-linker and potassium peroxydisulfate (KPS) as an initiator to radically polymerize AM in the mixed aqueous suspension of CNT and CNF. With a high aspect ratio and excellent mechanical performance, CNFs can effectively strengthen PAM and form high-strength PAM/CNF hydrogels [106]. The incorporation of CNTs endows the hydrogel with good electrical conductivity.

It has always been a great challenge to balance hydrogels' mechanical and electrical properties. Using conducting polymers as hydrogel networks tends to sacrifice some mechanical properties, making the hydrogel brittle. However, imparting conductivity to hydrogels through conductive fillers does not have this problem. Huang et al. [107] prepared SA-Zn ionic conductive hydrogel via radical polymerization using sodium alginate (SA), AA, AM ZnSO_4_ as raw materials, and APS as initiator. The conductive hydrogel conducts electricity mainly through conductive ions dissolved in water rather than conductive polymers. The introduction of ZnSO_4_ enhances the mechanical performance of the conductive hydrogel instead of weakening it due to the dynamic interaction of Zn^2+^ with ammonium/carboxyl groups. At the same time, the researchers also introduced functional monomers AA and AM under the premise of the existence of SA natural polymer, and the two were polymerized to build a double network structure, which further enhanced the stretchability of the hydrogel. Das et al. [108] devised a novel dual-network conductive hydrogel by introducing Fe^3+^ ions into poly(4-styrenesulfonic acid-methyl-uracilimidazole) chloride (PSS-MUI)/gelatin networks. To study the effect of different Fe^3+^ concentrations on the electrical conductivity and stretchability of hydrogels, the researcher team set up four groups of samples of pure PSS-MUI/gelatin hydrogels and PSS-MUI/gelatin hydrogels with 1 wt%, 2 wt% and 3 wt% Fe(NO_3_)_3_ (named PSUGF-1, PSUGF-2, PSUGF-3, respectively). With the increase of Fe(NO_3_)_3_ concentration, the electrical conductivity of the hydrogel is significantly improved (6.8, 9.1, and 10.3 S m^−1^ for PSUGF-1, PSUGF-2, and PSUGF-3, respectively), which is attributed to the increased number of Fe^3+^ cations and NO^3−^ anions inside the hydrogel. Meanwhile, the increase of Fe^3+^ concentration also improved the mechanical performance of the hydrogel owing to the network densification by metal–ligand interactions.

**Fig. 4:**
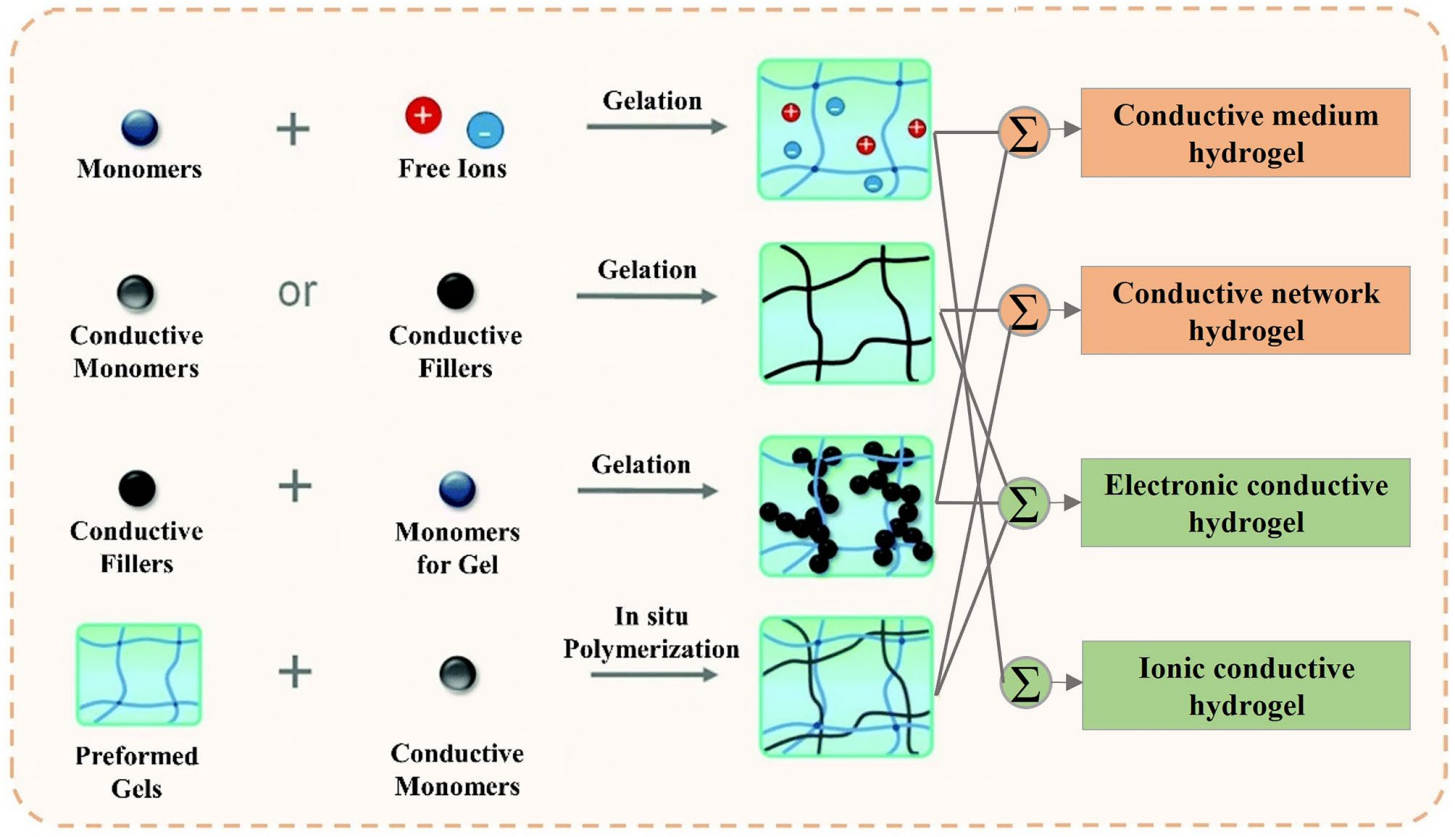
The main approach to produce conductive hydrogels and classification of hydrogels based on different principles [110]. Reproduced with permission. Copyright 2020, The Royal Society of Chemistry

In the preceding section, conductive hydrogels are categorized into conductive network hydrogels and conductive medium hydrogels according to their source of conductivity. From another point, conductive hydrogels can also be differentiated based on their conductive mechanisms, specifically electronic or ionic hydrogels [109] (Fig. 4). The former relies on electrons to conduct electricity and can be constructed by integrating conductive polymers, metallic nanomaterials, and carbon-based nanofillers. The use of conductive polymers and carbon-based nanofillers in electronic conductive hydrogels enables the creation of electron transport channels through conjugated structures. Metallic nanomaterials are also widely used to prepare conductive hydrogels due to their high electrical conductivity and high specific surface energy. On the other hand, ionic conductive hydrogels employ ions as conductive carriers that are free to move in solvents. Ionic conductive hydrogels are mostly transparent, while electronic conductive hydrogels are non-transparent owing to the opaque conductive components inside [110]. Ionic conductive hydrogels are widely utilized in wearable applications due to their high transparency and ability to create ionic gradients. However, one challenge with ionic conductive hydrogels is their propensity to experience ion diffusion into their surrounding environment [111]. In contrast, electronic conductive hydrogels possess superior biocompatibility and are thus better suited for use in bioelectronics and implant devices.

#### Self-adhesion

**Fig. 5:**
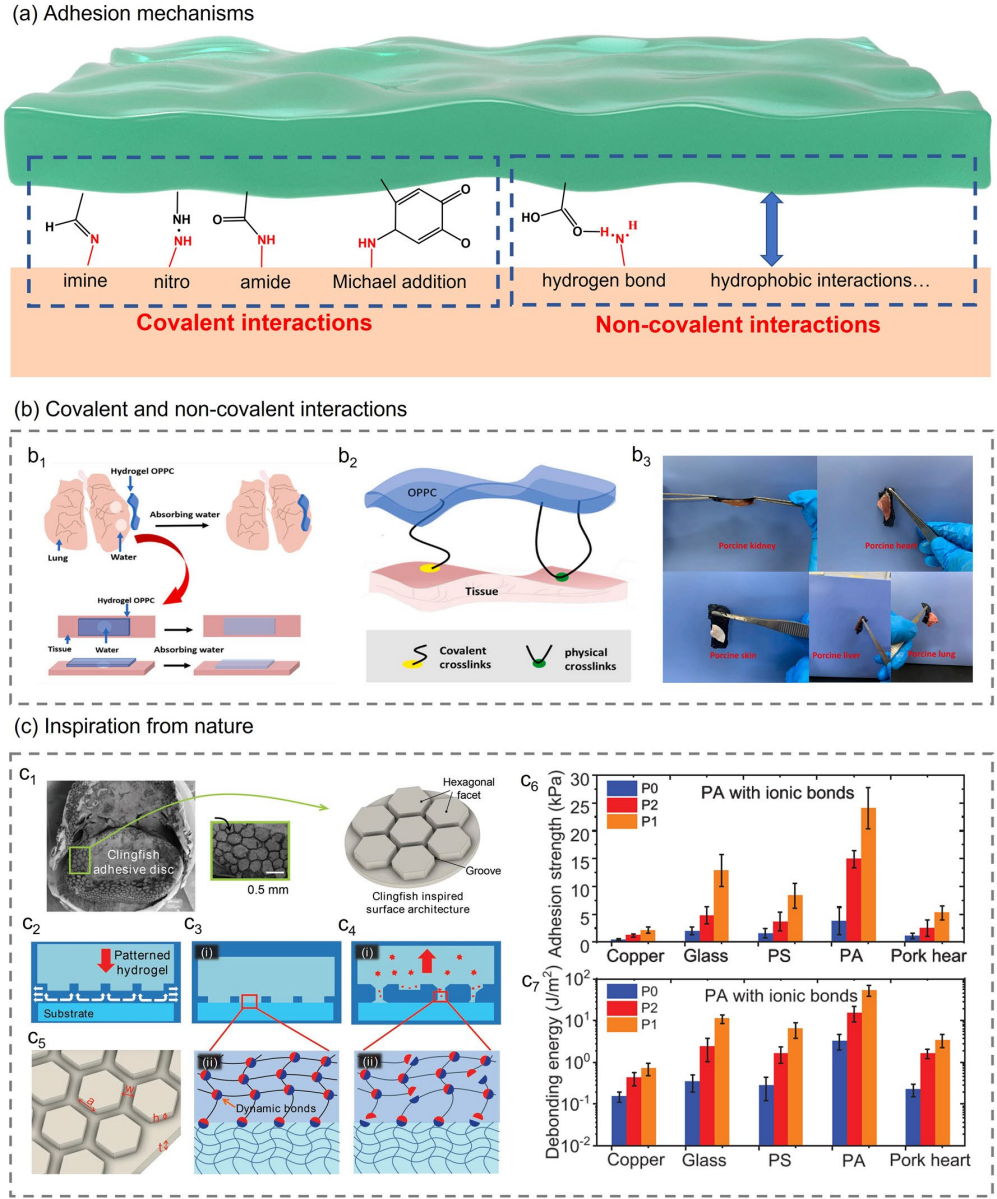
**a** Representative covalent/non-covalent linkages formed between self-adhesive hydrogel and substrate. **b** Achieving self-adhesion by introducing covalent or non-covalent bonds [119]. **b**_**1**_ The water on the surface of the wet organ is quickly absorbed by the hydrogel. **b**_**2**_ Adhesion mode of hydrogel OPPC. **b**_**3**_ OPPC hydrogels can easily adhere to the surface of various organs. Reproduced with permission. Copyright 2021, Elsevier. **c** Self-adhesive hydrogel inspired by nature [120]. **c**_**1**_ Inspired from the geometry of the adhesive discs of clingfish. **c**_**2**_ The grooves provide fast drainage channels. **c**_**3**_ The dynamic bonds between hexagonal facets and the substrates. **c**_**4**_ The breaking of dynamic bonds dissipates energy during deformation. **c**_**5**_ The geometric size of hexagonal facets. **c**_**6**_ Underwater adhesion strength, calculated from the ratio of the debonding peak force to the nominal surface area of the samples. (**c**_**7**_) Underwater debonding energy per unit area, calculated from the ratio of the area under the force–displacement curves to the nominal surface area of the sample. Sample P0 does not contain hexagonal facets, sample P1 and P2 have hexagonal facets with the length of 0.875 and 1.75 mm, respectively. Reproduced with permission. Copyright 2018, John Wiley and Sons

In the application scenario of wearable health monitoring, especially in organ and epidermal patch monitoring, self-adhesion is one of the most essential characteristics. Good self-adhesion can prevent the device from falling off and improve performance stability [112]. In addition, when used in wound monitoring, self-adhesive hydrogels can promote tissue repair by establishing interactions with surrounding tissue while preventing bacterial infection and blood loss [113–115]. The self-adhesion of hydrogels is mainly achieved by introducing covalent or non-covalent bonds between the hydrogel and the adhesive interface [116] (Fig. 5a). Many functional groups in hydrogels, such as hydroxyl, ether, amino, carboxyl, or catechol groups, can react with functional groups on the surface of tissues or organs to form imines, amides, or other covalent bonds. Non-covalent interactions are formed through hydrogen bonds, hydrophobic interactions, cation-Π interactions, and mechanical interlocking between the hydrogel and specific surface.

Covalent interactions are usually strong but irreversible, while non-covalent binding is weak but reversible. Thus, hydrogels with non-covalent adhesion mechanisms can be repeatedly attached and detached [117]. Chen et al. [118] designed a novel, intrinsically adhesive THBC hydrogel with unique “triple hydrogen bonding clusters” (THBCs) structure. Its high adhesion is mainly attributed to dense hydrogen bonds and the special equal load sharing (ELS) structure. The higher bond density provides more energy dissipation sites. The “Load-Sharing” Effect of the THBCs enhances the interfacial toughness under the same bond density. A peeling test was conducted to measure the maximum attachment energy. The result indicated that the maximum attachment energy increases with the increase of *N*-[tris(hydroxymethyl)methyl]acrylamide (THMA) content in the hydrogel, and the self-adhesive hydrogel with 80% THMA content has a maximum attachment energy of 422 J m^−2^. Adhesive interfaces based on non-covalent interactions are usually unstable. The adhesion of the hydrogel interface can be effectively improved by introducing covalent and non-covalent interactions. For example, Ren et al. [119] proposed an adhesion-enhancing hydrogel based on the above covalent and non-covalent synergistic strategy. The double-network OPPC hydrogel with excellent adhesion was prepared using O-carboxymethyl chitosan (O-CMCS) and PVA as raw materials, a supramolecular (PCD-CHO) with multiple aldehyde groups and borax as the cross-linking agent of the first cross-linked network and the second cross-linked network, respectively. When the hydrogel is attached to a wet organ, it can quickly absorb water, expands rapidly and adheres to the organ's surface (Fig. 5b_1_). The self-adhesion of OPPC hydrogel to tissues was mainly derived from abundant carboxyl groups from O-CMCS and aldehyde groups from PCD-CHO. The adhesion mode of hydrogel OPPC is shown in Fig. 5b_2_. The good hydrophilicity of the carboxyl group enables the OPPC hydrogel to form hydrogen bonds with the hydrophilic group on the skin surface. On the other hand, the aldehyde group provided by PCD-CHO reacts with the amino group of the skin surface protein through a Schiff base reaction to form a solid chemical covalent connection. Under the synergistic effect of covalent bonding and non-covalent bonding, the OPPC hydrogel exhibits excellent adhesion properties to various tissues and is expected to be applied to the monitoring of organ deformation in the future (Fig. 5b_3_).

In addition to studying the adhesion mechanism of hydrogels, researchers also tend to take inspiration from nature. Clingfish in nature exhibit rapid and reversible adhesion to various underwater surfaces. Rao et al. [120] analyzed the adhesive disc of clingfish. On the adhesive disc, they found many hexagonal features separated by interconnecting grooves, which provide fast drainage channels and ensure the excellent adhesion of the adhesive disc (Fig. 5c_1_–c_2_). Inspired by the clingfish, Rao et al. constructed similar hexagonal facets on hydrogel surfaces. The surface-engineered hydrogel has fewer defects, and the columns are independent, which can effectively delay the initiation and propagation of cracks at the interface (Fig. 5c_5_). Furthermore, the charge-balanced polyampholyte (PA) hydrogel used to prepare the surface microstructure contains a large number of dynamic bonds. As shown in Fig. 5c_3_–c_4_, these dynamic bonds not only form reversible bridges at the interface but also dissipate energy during deformation, which delays the adhesive's debonding. An underwater probe tack test was carried out to obtain the underwater adhesion strength of the surface-engineered tough hydrogels with dynamic bonds on different substrates (Copper, glass, polystyrene, polyampholyte and pork heart). The experiment was repeated on samples P0, P1 and P2, respectively. Among them, sample P0 is a flat hydrogel that does not contain a hexagonal surface structure, and its thickness is the same as that of samples P1 and P2. Sample P1 and P2 have hexagonal facets with the same height (*h*), groove width (*w*) and total thickness (*h* + *t*), and lengths (a) of 0.875 and 1.75 mm, respectively. Figure 5c_6_, c_7_ shows that sample P1 on the PA substrate has the most robust adhesion, and its adhesion strength and debonding energy can reach about 25 kPa and 50 J m^−2^. In addition to imitating the adhesive disc of clingfish, researchers also designed various self-adhesive hydrogels inspired by the adhesion mechanism of mussels. Due to the presence of catechol of 3,4-dihydroxy phenyl-l-alanine (DOPA) and amine groups from lysine amino acids inside mussel foot protein, marine mussels can adhere to the surface of various substrates. Han et al. [121] added polydopamine (PDA)-modified carbon nanotubes (CNTs) into the polyacrylamide-poly(acrylic acid) (PAM-PAA) polymer network and introduced glycerol–water binary solvent system through a solvent replacement strategy to prepare GW-hydrogel with good tissue adhesion. Compared with the pristine CNT-incorporated GW-hydrogel, the PDA-CNT-incorporated GW-hydrogel had stronger adhesion, and its highest adhesion strength to porcine skin was 57 ± 5.2 kPa. In addition, the adhesion of GW hydrogel is also affected by glycerol content. Its adhesion strength increased from 19 to 57 kPa when Gvol% increased from 12.5 to 50 vol%. The addition of PDA-CNT enhanced the hydrogel's electrical conductivity and mechanical properties, and the glycerol-water binary solvent enabled the hydrogel to maintain long-term stability in harsh environments such as cold and high temperatures. Given the above-mentioned numerous advantages, the mussel-inspired hydrogel is a promising self-adhesive bioelectronic material that can be applied in various complex environments. In general, the introduction of covalent/non-covalent adhesion mechanisms and the design of adhesion interface morphology are essential to impart hydrogel adhesion. The introduction of self-adhesive properties dramatically expands the application breadth of composite hydrogels. Composite hydrogels with self-adhesive properties have broad application prospects in wearable electronic devices, electronic skin/tattooing, and tissue repair and regeneration.

#### Self-healing

Wearable sensors are vulnerable to collisions or scratches during long-term wearing, which can lead to device damage or even functional failure [122]. Hydrogel materials with self-healing properties can rapidly and autonomously heal after damage and thus is suitable for preparing impact-resistant wearable devices [123]. Exploiting hydrogels with self-healing properties to prepare wearable devices can significantly improve the durability and lifetime of the devices.

**Fig. 6:**
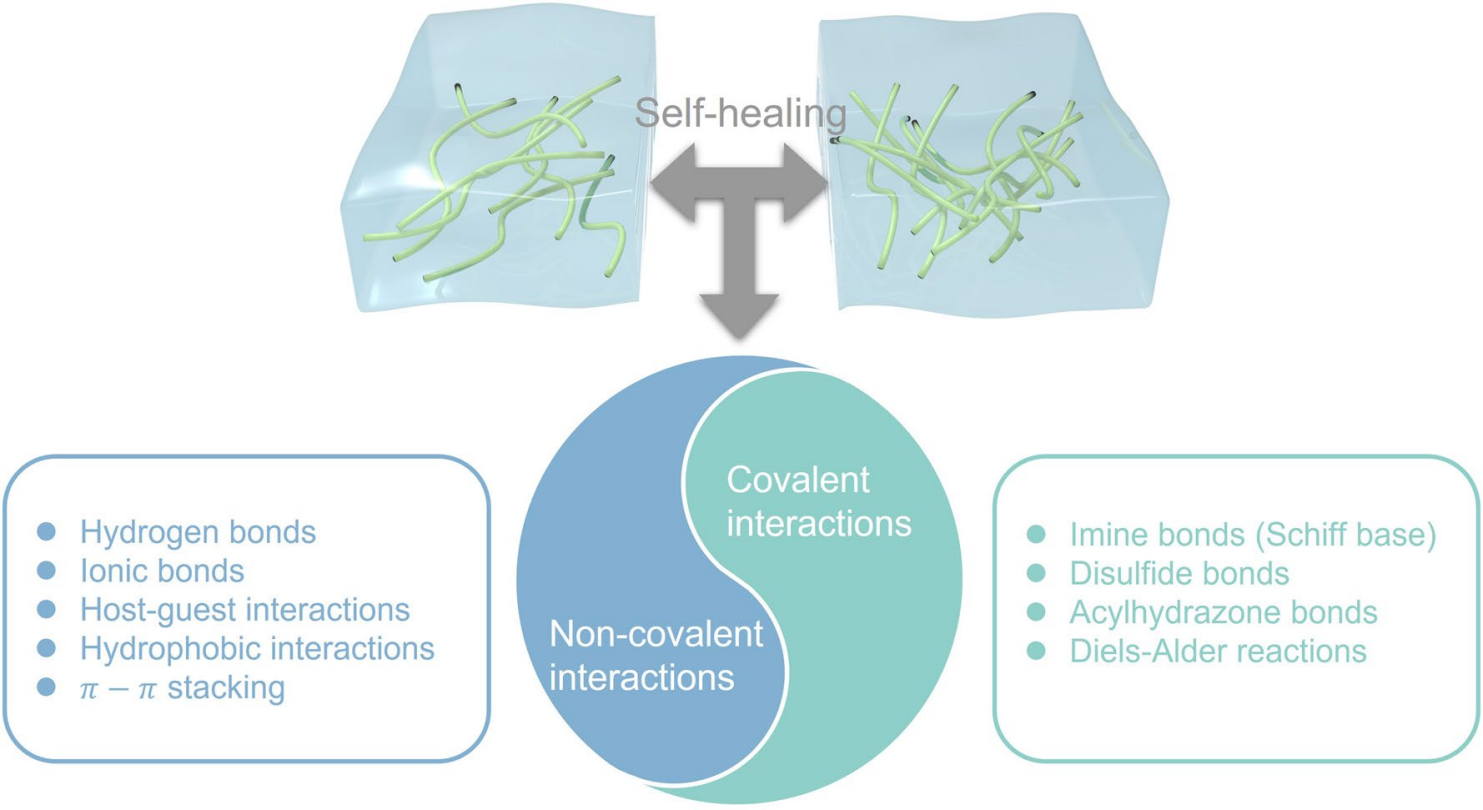
Summary of self-healing mechanisms

The self-healing properties of hydrogels are reliant on their internal reversible cross-linking mechanism [124] (Fig. 6). When the hydrogel is subjected to destructive operations such as cutting or impact, the reversible cross-linking bonds will be significantly destroyed, and cracks will occur. However, once the fractured surfaces are pressed together, the functional groups at the cross-section can re-establish reversible connections, thereby realizing rapid and autonomous healing of the hydrogel. Interactions such as hydrogen bonds, ionic bonds, host–guest interactions, hydrophobic bonds, and dynamic covalent bonds, which can be broken and reformed, are often introduced into hydrogels as internal driving forces for healing. Das et al. [108] proposed a novel DN hydrogel with outstanding self-healing ability. The PSUGF hydrogel consists of PSS-MUI, gelatin, Fe^3+^, and contains a large amount of dynamic hydrogen bonding and ionic and metal coordination interactions. These multiple reversible interaction mechanisms endowed hydrogels with strong adhesion, self-healing property and rapid electrical performance recovery. The researchers performed tensile tests on the original PSUGF hydrogel and the hydrogel after different healing times. After 2 h of healing time, the hydrogel recovered its original mechanical properties. Its tensile-strain curve almost entirely coincides with that of the pristine hydrogel, and the tensile strength reaches about 97% of the measured value of the pristine hydrogel. In addition, the researchers performed five cycles of cutting and healing at the same location on the hydrogel and recorded the ionic conductivity after each cycle. The ionic conductivity of five cycles does not change much and is the same as that of the pristine hydrogel. The above results indicated that under the synergistic effect of multiple self-healing mechanisms, the PSUGF hydrogel exhibited outstanding self-healing properties.

In addition to hydrogen and ionic bonds, host–guest interaction is also one of the critical self-healing mechanisms of hydrogels. The process by which host–guest molecules selectively combine to constitute supramolecules via non-covalent interactions is known as host–guest interaction. The non-covalent linkages between these supramolecules are often reversible and can be exploited as the driving force of self-healing. A self-healing hydrogel based on host–guest interaction strategy was proposed by Kakuta et al. [125]. The hydrophobic guest monomers (*n*-butyl acrylate (nBuAc) and *N*-adamantane-1-yl-acrylamide (AAm-Ad)) were dissolved in aqueous solutions of acrylamide-CDs (6-AAmCDs) to form inclusion complexes. Supramolecular hydrogels were successfully prepared by homogeneous radical copolymerization of inclusion complexes and AM. To evaluate the self-healing ability of supramolecular hydrogel, the adhesive strength of αCD–nBu gel and βCD–Ad gel were quantitatively analyzed via wedge strain compression test. First, the initial stress strength (*S*_0_) of each gel was determined by a rupture experiment. The researchers then recorded the surface bond strength (*S*_1_) at different healing times and calculated the recovery ratio of the adhesive strength (*S*_1_/*S*_0_). The experimental results showed that compared with *α*CD–nBu gel, βCD-Ad gel has better healing efficiency and can recover to its original strength.

Unlike the above-mentioned non-covalent interactions such as hydrogen bonds, ionic bonds, and host–guest interactions, dynamic covalent bonding can behave as reversible as non-covalent physical bonds or permanent like conventional covalent bonds depending on the conditions [126]. For example, Deng et al. [127] synthesized a new self-healing hydrogel containing two dynamic bonds—acylhydrazone and disulfide bonds. In an acidic environment, the hydrogel repairs damage through acylhydrazone exchange, while in an alkaline environment, the hydrogel self-heals through disulfide exchange. In a neutral environment, because both acylhydrazone and disulfide bonds are kinetically locked, the hydrogel cannot self-heal. However, adding catalytic aniline during preparation can accelerate the acylhydrazone exchange reaction, enabling the hydrogel self-healing in a pH = 7 environment. Although the dynamic polymer hydrogel proposed by Deng et al. [128] has outstanding healing properties, it still relies on the change of environmental pH to promote healing and thus is not actually "self-healing hydrogel". Afterward, Chen et al. propose a self-healing hydrogel based on dynamic covalent bonds by taking advantage of the spontaneous reaction between functional groups at the tangent surface of the hydrogel. Once the damaged surfaces of Ngel-Odex-ADH hydrogels are in contact with each other, the aldehyde groups of Odex can react spontaneously with the amino groups of Ngel and hydrazides of ADH, respectively, achieving spontaneous and rapid healing of the damaged area.

The self-healing properties of hydrogels mainly rely on their internal dynamic reversible bonds. These weak connections usually lead to the degradation of hydrogel's mechanical properties. At present, researchers are trying to adopt various cross-linking mechanisms to enhance the toughness of hydrogels. Among them, nanocomposite [129], chemical and physical cross-linking hybrid [130] and interpenetrating double network structure [131] are common strategies for hydrogel toughening. Hydrogels with self-healing properties and mechanical toughness can be obtained through proper modification of hydrogel engineering and are expected to be exploited in wearable devices to improve durability and life span.

#### Biocompatibility

Biocompatibility refers to the absence of interaction between a material and the human body or the degree of tolerance exhibited by the human body when exposed to such interactions, which may result in a range of complex biological, physical, and chemical reactions [132, 133]. Wearable health monitoring applications necessitate prolonged contact of the device with the human body, thereby putting more demand on the biocompatibility of its constituent components. Hydrogels comprise a three-dimensional polymer network embedded with considerable water, possessing a structure similar to soft tissue. Therefore, hydrogel materials have natural advantages over other flexible materials. Through the rational structural and component design of hydrogels, the physical, chemical, and biological properties of innate tissues can be mimicked to improve their biocompatibility.

The biocompatibility of hydrogels is greatly influenced by the constituent materials employed. Collagen, hyaluronic acid, chitosan, and polyethylene glycol, among others, represent primary materials for producing biocompatible hydrogels [134]. For instance, Ravishankar et al. [135] successfully prepared biocompatible hydrogels through the ionic cross-linking of chitosan and alkali lignin. The cytotoxicity of chitosan-alkali lignin gel was studied by the MTT method and showed a very low cytotoxicity of 99 ± 2% and 114 ± 0.2%, respectively. The xerogels of chitosan-alkali lignin gels also had a low cytotoxicity of 99 ± 3%, which demonstrates its excellent biocompatibility. In addition, the alkali lignin ionic cross-linkers used in the hydrogel synthesis process come from natural renewable resources. The renewability and cheapness of raw materials greatly enhance the attractiveness of chitosan-alkali lignin hydrogels as wearable electronic materials. In addition to avoiding damage to the human body, biocompatible hydrogels can even promote tissue regeneration. When the hydrogel material is applied to the wound, the 3D porous structure inside the hydrogel can carry bioactive components (such as biomolecules, proteins, and growth factors) to control and promote tissue regeneration and development [136].

The biocompatibility of hydrogel materials is not only related to the constituent materials but also affected by the synthesis method. Some toxic substances may be introduced into the hydrogel during synthesis, resulting in decreased biocompatibility. For example, the chemical cross-linking method of hydrogel promotes the cross-linking of polymer chains by adding specific chemical cross-linking agents. However, these cross-linking agents usually have specific toxicity, so the amount of cross-linking agent, reaction temperature and reaction time must be precisely controlled to avoid inflammation or cytotoxicity caused by cross-linking agent residues [137]. The cross-linking of physical hydrogels does not require toxic cross-linking agents but forms a cross-linked network through reversible non-covalent interactions, leading to relatively poor stability [138]. Compared with hydrogels synthesized by common chemical or physical methods, irradiation synthesis uses high-energy rays to trigger cross-linking reactions instead of cross-linking agents and initiators, thus possessing uniform cross-linking points and higher chemical stability [51].

## Health Monitoring Applications

### Physiological State Monitoring

As the pace of life accelerates, most people's body systems are severely out of balance due to long-term intensive work or study and lack of rest and exercise. The rapidly increasing levels of chronic illness and diminished well-being are challenging the public health system [139]. With increasingly prominent social health issues, people begin to pay attention to their health status, and the market demand for wearable health monitoring devices is expanding. Physiological state monitoring, as a crucial part of health monitoring, is attracting more and more attention. The physiological state is often used to describe the operating conditions of various life activities and functions within an organism and is a facile way to reflect human health conditions. Smart composite hydrogel-based wearable sensors with flexibility, stretchability, biocompatibility and other properties are suitable for monitoring human physiological signals. Smart composite hydrogel-based wearable sensors for physiological state monitoring and other health monitoring applications with different sensing mechanisms are summarized in Table 3 at the end of Section 3. Users can evaluate their physiological state by recording various physiological signals such as blood glucose, pulse, respiration, body temperature and so forth, which are significant to health management, disease prevention and control.

#### Sweat Monitoring

The main component of sweat is water, accounting for about 99%, in addition to a small number of nitrogenous compounds such as amino acids and urea, and metal or non-metal ions such as K^+^, Na^+^, and Cl^−^ [140]. The products of human metabolism are often excreted together with sweat through sweat glands. When a person's physiological state is out of balance or sick, some components of sweat will also change accordingly [141, 142]. Therefore, real-time sweat monitoring is an effective way to learn about the user's health status. During sweat monitoring, sensors need to be applied on the surface of human skin, which requires the sensing material to be flexible, stretchable and biocompatible. Smart composite hydrogel regulated by hydrogel engineering meets the demand quite well, and there are many reports concerning composite hydrogel-based sweat sensors worldwide.

**Fig. 7:**
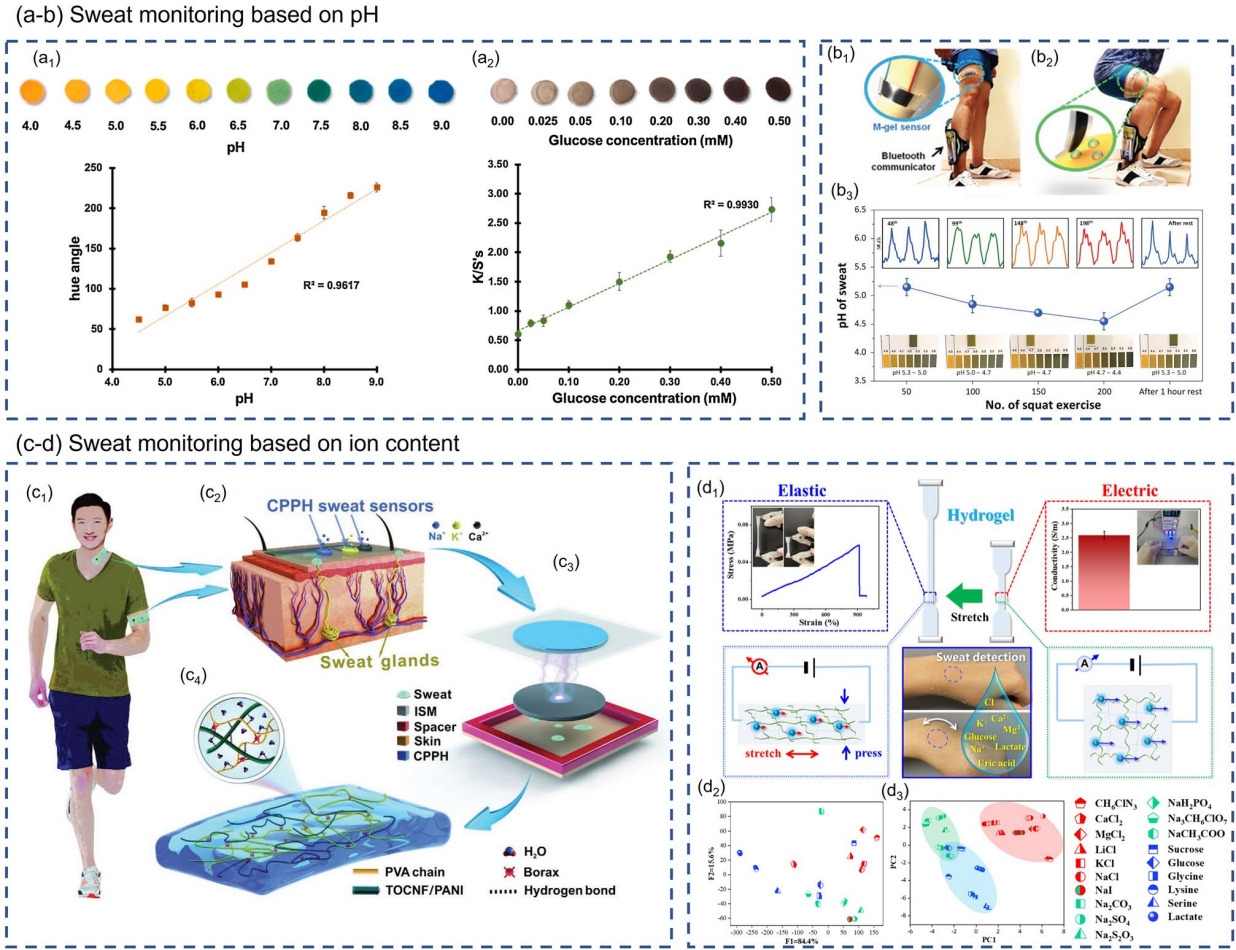
Composite hydrogel-based wearable sweat monitoring. **a**, **b** Composite hydrogel-based pH sensors for wearable sweat monitoring [143, 148]. **a**_**1**_ Standard color chart and a calibration plot of the BC/CMC-based pH sensor over a pH range of 4.0–9.0. **a**_**2**_ Standard color chart and a calibration plot of BC/CMC-based glucose sensor in a concentration range of 0.00–0.50 mM glucose. Reproduced with permission. Copyright 2021, Elsevier. **b**_**1**_ Before exercise, M-hydrogel sensor and bluetooth communicator were installed on the thigh and ankle, respectively. **b**_**2**_Sweat makes contact with M-hydrogel sensor during squat exercise. **b**_**3**_ Comparison of pH values measured by M-hydrogel sensor and pH test paper. Reproduced with permission. Copyright 2021, John Wiley and Sons. **c**, **d** Composite hydrogel-based sensor for the detection of ionic content in sweat [149, 150]. **c**_**1**_ Real-time monitoring of ion concentrations in human sweat during exercise. **c**_**2**_ CPPH sweat sensor is placed on cutaneous sweat glands to detect and quantify Na^+^, K^+^, and Ca^2+^. **c**_**3**_ The structure of the CPPH sweat sensor. **c**_**4**_ Internal structure diagram of CPPH. Reproduced with permission. Copyright 2022, John Wiley and Sons. **d**_**1**_ An elastic-electric coefficient-sensitive hydrogel sensor for monitoring and identifying sweat. **d**_**2**_ LDA result of elastic − electric coefficient sensitivity shows a clear clustering of the 19 analytes and 100% correct classification. **d**_**3**_ PCA graphs show the 19 analytes are successfully classified into three categories, i.e., anions, cations, and organic compounds. Reproduced with permission. Copyright 2022, American Chemical Society

**Sweat Monitoring Based on pH.** Siripongpreda et al. [143] successfully prepared a re-swellable and biocompatible hydrogel by directly depositing negatively charged polyelectrolyte, carboxymethyl cellulose (CMC) into bacterial cellulose (BC) matrix. The addition of hydrophilic CMC effectively prevents the collapse of the BC fiber structure during drying, endowing the BC/CMC hydrogel with better re-swellability than the BC hydrogel. Among all sensing technologies for monitoring biomarkers, colorimetry is the most common one due to its advantages of high sensitivity, simple method and easy operation [144–146]. The researchers fabricated the colorimetric pH sensor by soaking the BC/CMC hydrogel in a specific pH indicator solution. As shown in Fig. 7a_1_, the BC/CMC hydrogel-based pH sensor exhibits good linearity in a wide range of pH 4.0–9.0 and can well monitor pH changes in sweat (pH 4.0–9.0 for sweat [147]). Moreover, BC/CMC hydrogels can also be exploited as colorimetric glucose sensors after adding an enzyme mixture and potassium iodide (KI) to monitor the glucose level in sweat. The H_2_O_2_ produced by the enzymatic reaction between glucose oxidase (GO_x_) and glucose in sweat will oxidize KI, causing the sensor's color to change from yellow to brown (as shown in Fig. 7a_2_). The BC/CMC hydrogel-based glucose sensor exhibits an extremely low limit of detection (25 μM) and a wide monitoring range (0.0–0.5 mM). Lee et al. [148] also carried out much research work on wearable sensors for sweat detection. The researchers embedded MXene (Ti_3_C_2_T_*x*_) into the PAA/PVA hydrogel composite to obtain M-hydrogel, which is very sensitive to pH changes due to the cation-selective surface conductance in the vicinity of negatively charged MXene sheets. The pH sensor fabricated from M-hydrogel exhibits a fast response. Its resistance reaches a maximum change in a short time (2 data points ≈ 50 ms) after contact with the PBS (phosphate buffer saline) solution. When the PBS solution was removed from the M-hydrogel surface, its resistance gradually recovered to its original value, proving the reversibility of the M-hydrogel sensor. Lee et al. also applied M-hydrogel to the subject's thigh to further investigate its ability to monitor the sweat's pH. After 50, 100, 150, 200 squats followed by 1 h of rest, they measured the pH of the sweat using pH test paper and the change in the resistance of the hydrogel sensor (as shown in Fig. 7b). In this way, the relationship between the pH of actual sweat and the electromechanical response of M-hydrogel could be established.

**Sweat Monitoring Based on Ion Content.** In addition to pH, the ion content of sweat is also closely related to human health. Qin et al. [149] designed a TENG-based (TENG, triboelectric nanogenerator) sensor based on cellulose conductive composite hydrogel, which can be used to detect various ions in sweat. The structure of the sensor is shown in Fig. 7c. The TOCNF (2,2,6,6-tetramethylpiperidine-1-oxyl radical (TEMPO)-oxidized CNFs)/PANI-PVA hydrogel (CCPH) electrode is wrapped by PDMS and then assembled with an ion-selective membrane to form a self-powered sweat sensor. PDMS and ISM act as negative triboelectric and positive triboelectric materials, respectively, to realize the contact electrification of TENG. The ISM module is also responsible for separating specific sweat ions such as Na^+^, K^+^, and Ca^2+^. Due to biomechanical vibrations, ISM and PDMS undergo periodic contact-separation cycles, resulting in unique electrical signals. When the human body sweats, specific ions in the sweat will pass through and stay on the surface of the ISM, causing the potential change on the surface of the ISM to eventually lead to the change of the electrical signals. According to the Hofmeister effect, different salts have different effects on the solubility of polymers. Based on this strategy, Shen et al. [150] prepared PVA-ATMP (poly(vinyl alcohol)-amino trimethylene phosphonic acid) hydrogel sensor for sweat detection. The electrical and mechanical properties of PVA-ATMP hydrogels will vary with cosolvent type and concentration. When the hydrogel is added with well-hydrated kosmotropes, it promotes the aggregation of hydrophobic solutes and causes the entanglement of polymer chains. By contrast, the addition of weak-hydrated chaotropes suppresses hydration and induces the polymer chain slack [151–153]. Changes in the polymer network structure not only affect the mechanical properties of hydrogels but also impact the electrical properties by changing the conductive channels. The researchers established a dual-parameter model based on elastic and electrical sensitivity and constructed an elastic-electric coefficient-sensitive hydrogel sensor for monitoring and identifying sweat (Fig. 7d_1_). The researchers exploited 19 cosolvents to simulate changes in the composition of sweat, including six cations, seven anions and six organic chemicals. With elastic sensitivity and electrical sensitivity as variables, linear discriminant analysis (LDA) and principal component analysis (PCA) were used to distinguish cosolvent clusters containing different ions. The LDA results in Fig. 7d_2_ demonstrate that 100% correct classification of all 19 analytes was achieved using elastic − electric coefficient sensitivity. The PCA results in Fig. 7d_3_ show that the 19 analytes can be classified into three categories: anions, cations, and organic chemicals. A series of experiments demonstrate the feasibility of ATMP-PVA hydrogel for wearable sweat monitoring.

#### Respiratory Monitoring

Respiration is the process of gas exchange between the body and the external environment. Humans maintain the function of cells throughout the body by inhaling oxygen and expelling carbon dioxide [154]. Therefore, respiration and health are inseparably related, and respiration often reflects the level of human health. Currently, there are mainly two breath detection methods based on smart composite hydrogel sensors. One is to use smart composite hydrogel-based strain sensors to monitor the contraction and relaxation of the lung or thoracic cavity during respiration [155–157]. The second is to use smart composite hydrogel-based sensors to detect exhaled air directly and thereby monitor the respiratory status [158, 159].

**Fig. 8:**
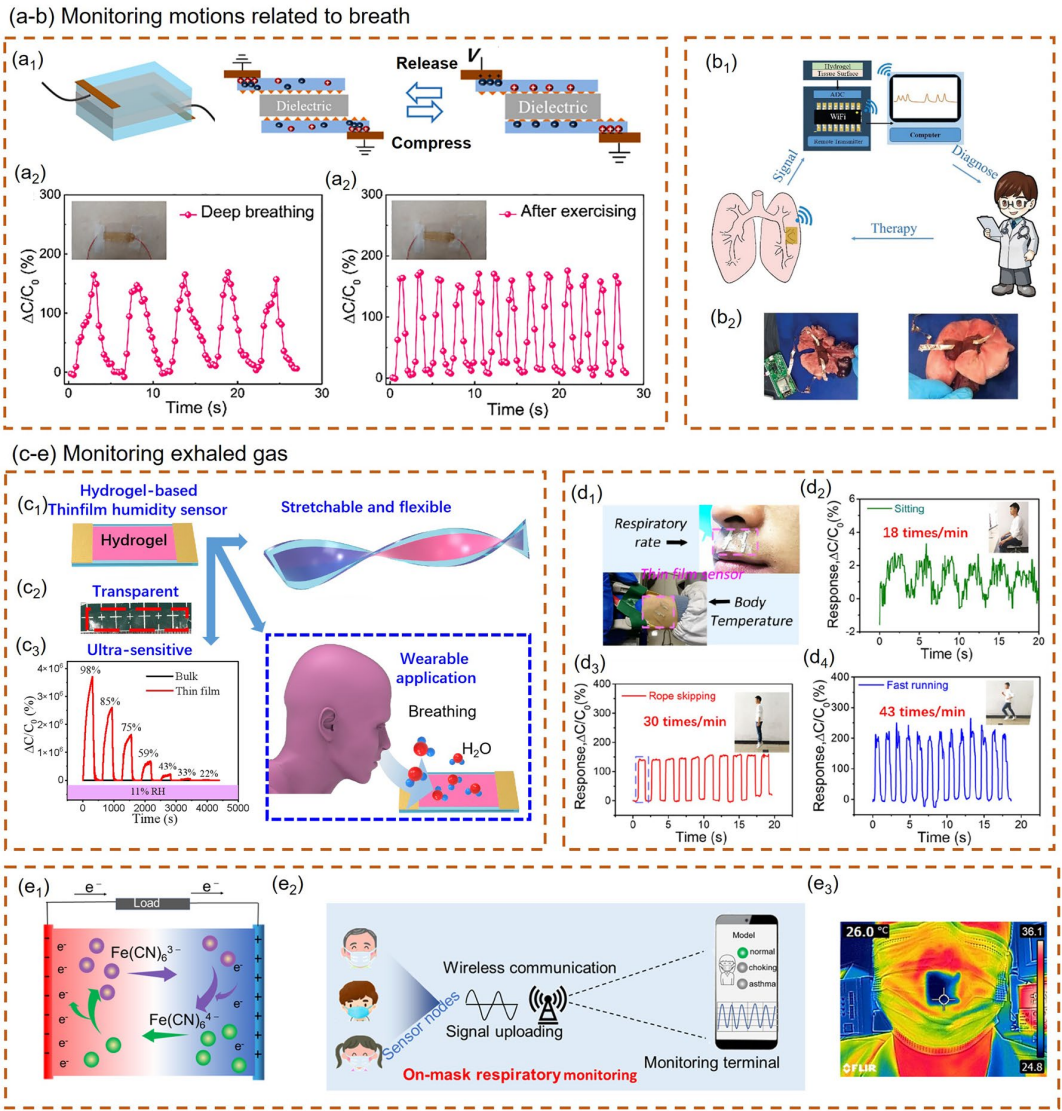
Composite hydrogel-based wearable respiratory monitoring. **a**, **b** Respiratory monitoring method by attaching hydrogel sensors to the surface of the chest cavity or lung for monitoring [161, 163]. **a**_**1**_ Schematic diagram of as-assembled capacitive pressure sensor. **a**_**2**_, **a**_**3**_ The sensor is attached to the wearer’s abdomen to record the change of capacitive signal during deep breathing and breathing after exercise. Reproduced with permission. Copyright 2021, Elsevier. **b**_**1**_ The respiratory monitoring data collected by the hydrogel patch is transmitted to the computer through a wireless transmitter for subsequent processing and analysis. **b**_**2**_ The respiratory signal is recorded by a hydrogel sensor attached to the rabbit lung and transmitted through wired (left) and wireless (right) communication. Reproduced with permission. Copyright 2014, Royal Society of Chemistry. **c**–**e** Respiratory monitoring method by monitoring exhaled gas [26, 165, 166]. **c**_**1**_ PAM/CA hydrogel film humidity sensor. **c**_**2**_ The white marks below can be seen through the PAM/CA hydrogel film, which proves the excellent transparency of the hydrogel film. **c**_**3**_ Comparative studies showed that the responses of these sensors are thickness dependent. Thin film hydrogel sensor has higher sensitivity than bulk hydrogel. Reproduced with permission. Copyright 2022, Springer Nature. **d**_**1**_ TFSS sensors are attached to the skin of the upper lip and wrist, respectively. **d**_**2**_–**d**_**4**_ Capacitance changes of sensor during sitting, rope skipping and fast running, respectively. Reproduced with permission. Copyright 2021, American Chemical Society. **e**_**1**_ Thermoelectric conversion mechanism of PVA/GEL thermogalvanic hydrogel. **e**_**2**_ Schematic diagram of on-mask respiratory monitoring system. **e**_**3**_ Infrared image of the wearing mask showing a visual Δ*T* between the gel sensor in the mask and ambient. Reproduced with permission. Copyright 2022, American Chemical Society

**Monitoring Motions Related to Breath. **The first method requires attaching hydrogel sensors to the surface of the chest cavity or lung for monitoring. Thus, the hydrogels used are usually self-adhesive and biocompatible. Xia et al. [160] proposed a polyacrylamide/chitosan (PAM/CS) hybrid hydrogel network, in which the PAM network is cross-linked by hydrophobic association and the CS network is formed by ionically cross-linking of carboxyl-functionalized multi-walled carbon nanotubes (c-MWCNTs). The efficient energy dissipation of the dynamic cross-linked network inside the hydrogel endows it with excellent flexibility, puncture resistance and self-healing capability. Moreover, PAM/CS hydrogel also possesses strong self-adhesion and can be applied to various material surfaces, including skin and organ surfaces. The sensor was attached to the chest cavity and exhibited different resistance values for different breathing modes, such as regular breathing, rapid deep breathing, and breathing hold. Wang et al. [161] successfully prepared a self-buckled PAM/alginate hydrogel (SPAH) via a stretching/competitively-coordinating/releasing (SCR) strategy. The high sensitivity (3.19 kPa^−1^), low LOD (Limit of detection) (25 Pa), and excellent durability (> 1400 cycles) derived from SPAH's unique wrinkled surface structure make it suitable for skin-inspired pressure sensing. The capacitive pressure sensor was fabricated by integrating SPAH samples with a dielectric layer (Fig. 8a_1_). Due to the unique wrinkled surface of SPAH, the SPAH pressure sensor will produce a large area change when subjected to external pressure, resulting in a huge capacitive response. Thus, the SPAH capacitive pressure sensor can precisely monitor tiny movements of human bodies, such as finger bending, speaking, and breathing. Figure 8a_2_–a_3_ shows the application scenario where the sensor is attached to the wearer's abdomen to record the change of capacitive signal during deep breathing and breathing after exercise.

The smart composite hydrogel-based sensors reported above indirectly monitor respiratory conditions by detecting the heaving of the chest or abdomen during breathing. This method which only monitors the subtle human motion caused by breathing without involving complex biochemical reactions, is characterized by a simple process and low cost. In the monitoring process, the sensor will record the strain signal generated by the collision or inadvertent action, seriously interfering with the respiratory monitoring. Therefore, some research teams try directly attaching the sensor to the lungs to monitor breathing, which can effectively remove most of the strain signals generated by non-breathing behavior. Ling et al. [162] applied the prepared dialdehyde carboxymethyl cellulose/amino gelatin/polyacrylic acid (DCMC/AG/PAA) hydrogel strain sensor to the surface of the pig lung to simulate human respiration monitoring. The air pump and the medical pressure ball were used to simulate porcine lung breathing, respectively. The resistance value of the sensor changed periodically with the contraction and relaxation of the porcine lung, proving that the composite hydrogel-based strain sensor has great potential for clinical pulmonary respiratory monitoring. Pei et al. [163] also proposed a PDA–clay–PSBMA hydrogel that is adhesive enough to be attached to the lungs and is highly sensitive to strain for pulmonary motion monitoring. The research team applied the hydrogel patch to rabbits' lungs to simulate its dynamic monitoring. The rabbit's lung was punctured with a needle to simulate a pneumothorax. The adhesive properties of the hydrogel enable the rapid closure of lung wounds. Each time air is pumped into the lungs, the hydrogel changes its electrical resistance as it is stretched and returns to its original state as the lungs relax. During the breath simulation, the hydrogel patch generates a series of electrical signals that change with the movement of the lungs. The respiratory monitoring datas collected by the hydrogel patch are transmitted to the computer through a wireless transmitter for subsequent processing and analysis (Fig. 8b).

#### Pulse and Heartbeat Monitoring

Due to the influence of cardiovascular changes, pulse and heartbeat are not precisely equivalent [167]. Nevertheless, both of them carry valuable information about cardiovascular disease and are often used to diagnose diseases such as hypertension, arteriosclerosis, and cardiomyopathy [168–170]. Real-time monitoring of pulse and heartbeat has tremendous implications for preventing cardiovascular disease. The current wearable pulse and heartbeat monitoring based on smart composite hydrogels is mainly realized by strain sensing.

**Monitoring Exhaled Gas.** Applying composite hydrogel-based strain sensors to the lungs for respiratory monitoring can avoid the interference signals caused by collision or unintentional action. However, since there is no direct detection of the exhaled gas, this method cannot analyze the composition of exhaled gas. Hence, the accessible human physiological indicators are very limited. Moreover, this implantable measurement method requires complex operations to install or remove the device, which brings additional risks to users. Based on this actuality, many researchers have attempted to achieve non-invasive and non-implanted respiratory monitoring by monitoring exhaled gas. Zeng et al. [164] successfully prepared transparent, highly flexible and multifunctional starch/PAM double-network hydrogels through a facile one-step strategy. The prepared hydrogel showed not only fast self-adhesivity and high flexibility but also high sensitivity to humidity (35–97% RH), allowing it to identify different lengths and intensities of the exhalation. Wu et al. [165] successfully prepared PAM/CA hydrogel films with different thicknesses by spin coating and constructed the hydrogel film humidity sensor (Fig. 8c). Comparative studies showed that the responses of these sensors are thickness dependent. Their sensing performance is unprecedentedly enhanced due to the miniaturization effect of the hydrogel and the enlarged surface area, which promote the adsorption and desorption of moisture. The thinnest (6.06 μm) hydrogel thin film humidity sensor exhibits an ultrahigh sensitivity of 78785.5%/% RH, and its response at 98% RH is 203,703 times higher than that of the bulk hydrogel. The hydrogel film is promising for monitoring small humidity changes during respiration due to its high humidity sensitivity. Wu et al. [26] also used a layer-by-layer spin-coating technique to design a hydrogel-based temperature sensor with a thin-film sandwich structure (TFSS). The device consists of PDMS film, PAM/CA hydrogel film, and PDMS film from top to bottom. Silver paste is deposited on both ends of the hydrogel film as electrodes. The PDMS film wrapped outside can effectively prevent moisture evaporation, increase heat transfer, and significantly improve the sensor's stability and response/recovery speed. The sensor can also monitor human respiration due to the temperature difference between exhaled air and the environment. The capacitance of TFSS immediately increases when exhaling and rapidly recovers when inhaling. Figure 8d_2_–d_4_ shows the capacitance changes of sensor during sitting, rope skipping and fast running, respectively. The respiration frequencies are calculated to be 18, 30, and 43 times/min through the period of capacitance change.

Although the sensors mentioned above can be attached to the chest, lungs or upper lip to achieve wearable respiratory monitoring, they all need to be powered by an external power source, which cannot be genuinely wearable. The sensor prepared by Li et al. [166] using thermogalvanic hydrogel can convert the heat energy in the exhaled gas into electrical energy, successfully realizing the self-power supply of the wearable sensor. The redox pair Fe(CN)_6_^3−^/^4−^ inside the PVA/GEL thermogalvanic hydrogel plays a critical role in thermoelectric conversion. Its thermoelectric conversion mechanism is shown in Fig. 8e_1_. Owing to the high temperature of the cathode, the oxidation reaction of Fe(CN)_6_^4−^ → Fe(CN)_6_^3−^ + e^−^ occurred on the cathode surface. This reaction injects electrons into the cathode electrode, causing the electrode potential to decrease. On the anode electrode, the lower temperature promotes the reduction reaction Fe(CN)_6_^3−^ + e^−^ → Fe(CN)_6_^4−^. During the reaction, electrons are induced from the anode electrode, increasing the potential of the anode electrode. The temperature gradient across the electrodes leads to the formation of a potential difference, enabling the sensor's self-powered function. Furthermore, the research team integrated the hydrogel sensor into the mask, sent the data to the terminal through wireless transmission, and finally identified the breathing pattern through frequency analysis (Fig. 8e_2_, e_3_). The work of Li et al. demonstrates the great potential and broad prospect of an integrated wearable system integrating detection, power supply system, and data transmission functions.

**Fig. 9:**
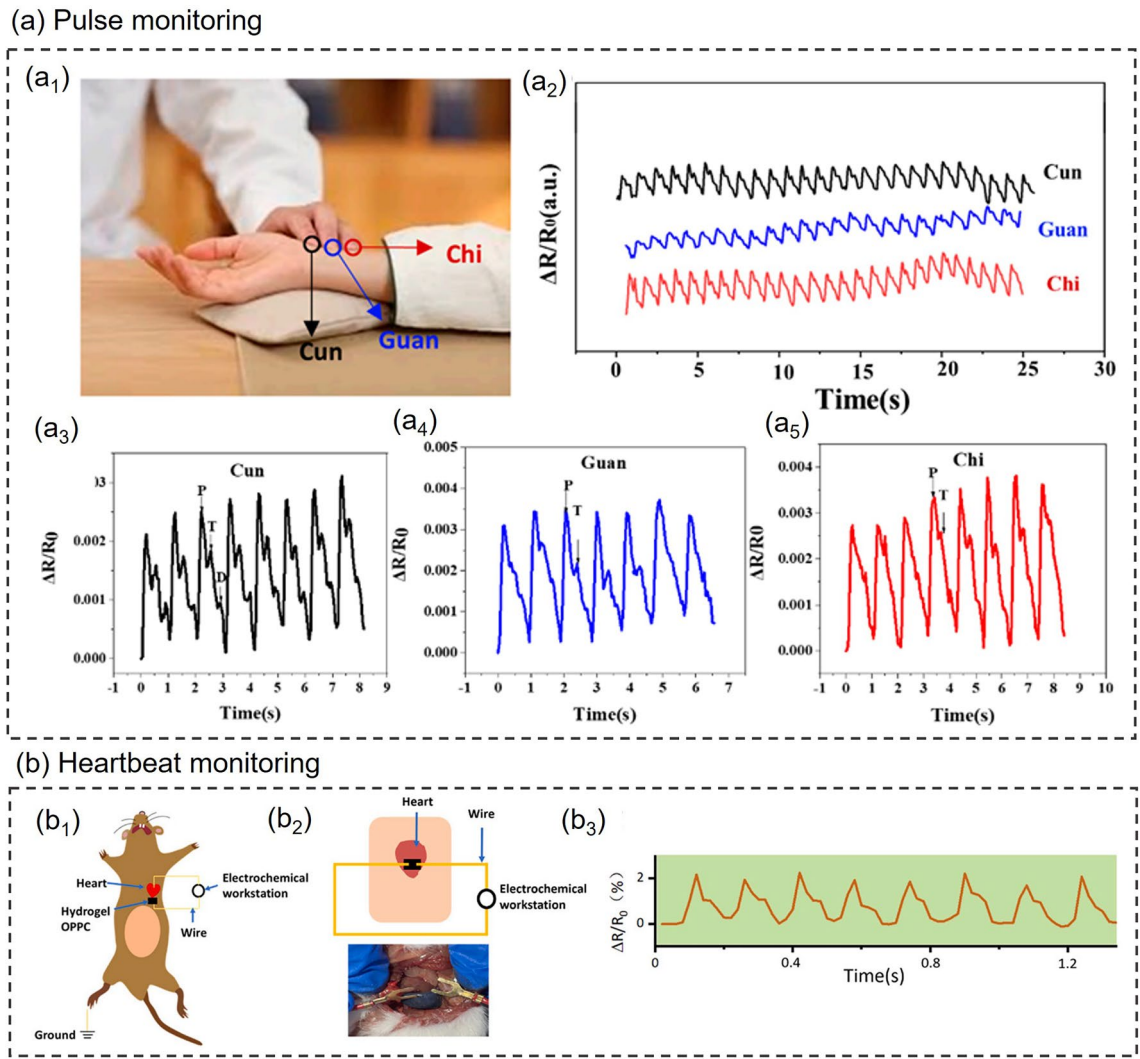
**a** Pulse monitoring [100]. **a**_**1**_ A photograph of TCM pulse diagnosis. **a**_**2**_ Cun, Guan and Chi pulse waveforms of a 25-year-old male subject. **a**_**3**_–**a**_**5**_ Measured typical pulse waveforms at the Cun, Guan, Chi positions, respectively. Reproduced with permission. Copyright 2014, American Chemical Society. **b** Heartbeat monitoring [119]. **b**_**1**_ Schematic illustration of the rat heart beating recorded by hydrogel OPPC as a sensor. **b**_**2**_ Schematic illustration of hydrogel OPPC attached to a mouse heart. **b**_**3**_ The electrical signals generated by the beating of the rat heart. Reproduced with permission. Copyright 2021, Elsevier

**Pulse Monitoring. **Zhang et al. [171] successfully prepared ultrastretchable M-hydrogel by mixing MXene (Ti_3_C_2_T_x_) nanosheets with a low-cost commercial hydrogel commonly used in toys, namely “crystal clay” which is composed of poly(vinyl alcohol) (PVA), water, and anti-dehydration additives. The M-hydrogel exhibits high stretchability (more than 3400%), excellent self-healing ability, self-adhesion and high strain sensitivity (Gauge Factor = 25) owing to the variation of interlayer distance of Mxene under external pressure. The research team successfully achieved human pulse monitoring by attaching the M-hydrogel-based mechanical sensor to the carotid artery of the neck of the test subject. Wang et al. [100] report a novel tough conductive hydrogel composed of interpenetrating PANI and poly(acrylamide-co-hydroxyethyl methyl acrylate) (P(AAm-co-HEMA)) networks. The as-prepared strain sensors made from this hydrogel are very sensitive even at deficient strain and thus are very suitable for monitoring weak vibrations such as pulse and voiceprints. The hydrogel-based strain sensor is mounted on the wrist to monitor the periodic pulse signal. The pulse rate recorded by the hydrogel-based strain sensor is consistent with the pulse rate obtained from the ECG of the same subject. Experiment results demonstrate the potential of hydrogel sensors for pulse monitoring. Shen et al. [172] developed a hydroxyethylidene diphosphonic acid (HEDP) assisted poly(vinyl alcohol) (PVA) composite hydrogel to achieve a high-performance stretch-sensitive sensor. Interestingly, the research team applied the PVA-HEDP hydrogel-based strain sensor to the three-finger palpation of Traditional Chinese Medicine (TCM). In TCM diagnosis, TCM doctor diagnoses patients by detecting the pulse at corresponding points on the radial artery defined as Cun, Guan and Chi positions, as shown in Fig. 9a_1_. Shen et al. used a three-channel PVA-HEDP hydrogel-based sensor array to replace the three fingers of the TCM doctor and recorded the pulse waves at the three positions of Cun, Guan, and Chi. As can be seen from Fig. 9a_2_-a_5_, the pulse waves recorded at the three positions have three typical peaks, namely percussion wave (P), tidal wave (T) and dicrotic wave (D), but the T and D wave signals at Guan and Chi positions are relatively weak. In Traditional Chinese Medicine, the pulses of Cun, Guan and Chi are exploited to assess the health status of patients. PVA-HEDP hydrogel strain sensor can provide real-time pulse information of Cun, Guan and Chi, which is helpful for accurate diagnosis of TCM.

**Heartbeat Monitoring. **Besides pulse, heart rate is also a crucial physiological indicator of health. At present, some researchers try to attach hydrogel strain sensors to the surface of the heart for implantable heart rate monitoring, but related research is still in the stage of animal experiments. Zhang et al. [173] prepared Alg-CNT hydrogel using carbon nanotubes (CNTs), alginate (Alg) modified with 3-aminophenylboronic acid (PBA) and dopamine (DA) as raw materials. The as-prepared Alg-CNT hydrogel showed rapid self-healing properties (30 s), excellent stretchability (500%) and self-adhesiveness to various substrates. The Alg-CNT hydrogel-based strain sensor is highly suitable for monitoring subtle expansion–contraction motions. The researchers tried applying the Alg-CNT hydrogel strain sensor to the heartbeat monitoring of mice. The mouse was first anesthetized and then subjected to a median sternotomy, and the hydrogel sensor was quickly adhered to the anterior surface of the heart. The Alg-CNT hydrogel-based strain sensor shows electrical signals that vary with the mouse’s heart rhythm. Ren et al. [119] fabricated a hydrogel sensor based on o-carboxymethyl chitosan (O-CMCS) and PVA for monitoring human and organ motions. The hydrogel has rapid self-healing ability with healing efficiency as high as 97–103% (in 15 s), good adhesion to human skin and wet organs, excellent antibacterial properties, cytocompatibility, and stretchability, hence it is very suitable for implantable organ monitoring. As shown in Fig. 9b, when the hydrogel OPPC was in contact with the surface of the mouse’s heart, the hydrogel quickly absorbed the moisture on the tissue surface and adhered firmly to it. The resistance value of the smart composite hydrogel sensor connected to the electrochemical workstation changes periodically with the mice's heartbeat, thereby converting the heartbeat signal into an electrical signal. Through this principle, it is expected to realize real-time monitoring of the human heart organ in the future.

#### Body Temperature Monitoring

The body temperature of ordinary people tends to be relatively stable, roughly around 37 °C [174]. Although body temperature varies with factors such as gender, age, environment, time, exercise, and mood, all these changes are within normal limits. A body temperature which exceeds the normal range often means that there is a problem with bodily functions. Body temperature is directly related to human health. Especially at present, COVID-19 is still raging worldwide, and fever happens to be a prominent symptom of pneumonia [175]. Therefore, monitoring body temperature has become an effective means to screen patients and prevent the spread of the coronavirus. Against this backdrop, the demand for wearable temperature sensors for continuous real-time monitoring is sharply increasing. There have been many reports on hydrogel-based temperature sensors worldwide [176–178]. The following section will mainly introduce the application of smart composite hydrogel-based temperature sensors in wearable body temperature monitoring.

**Fig. 10:**
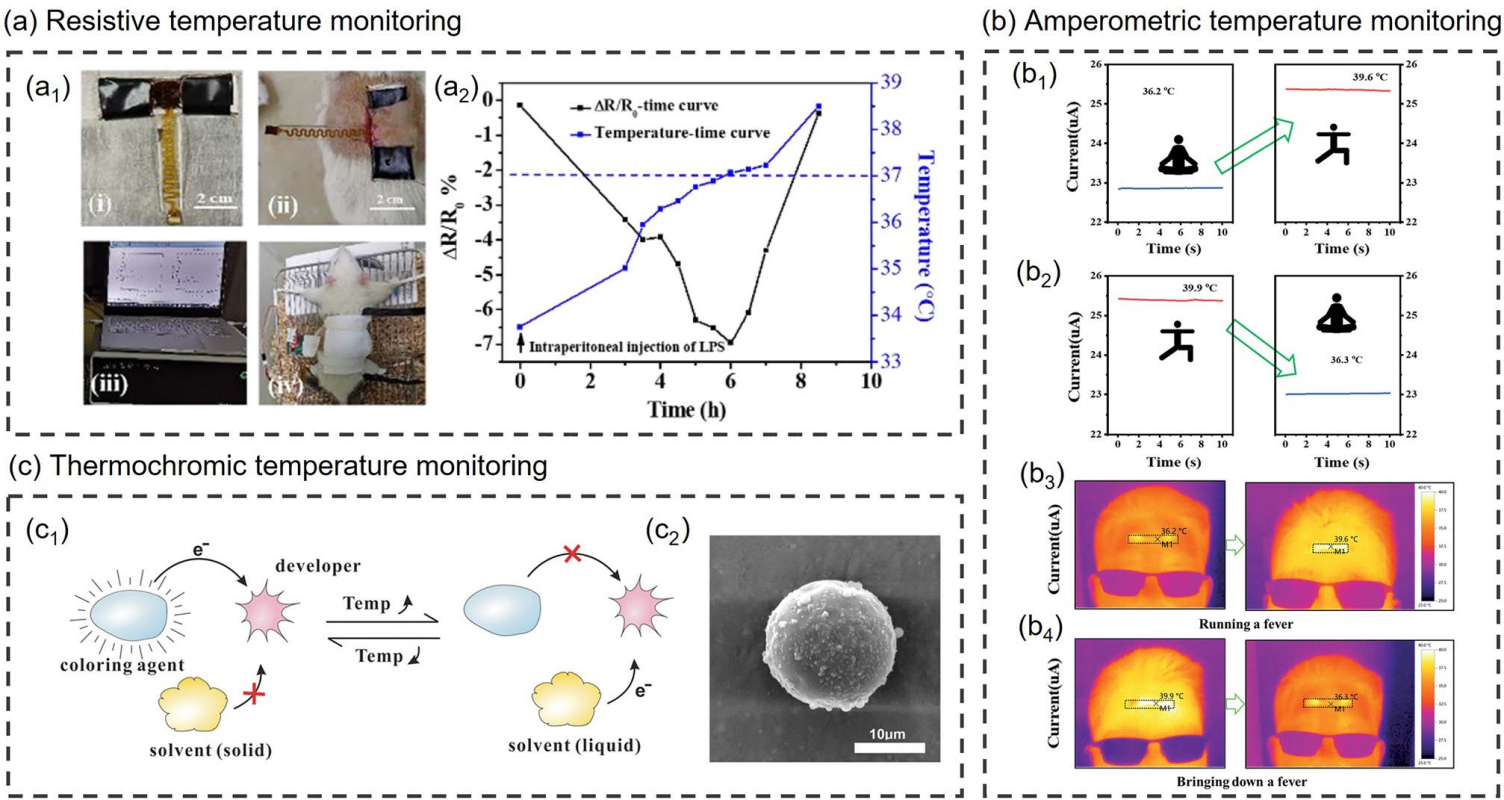
**a** Resistive temperature monitoring [179]. **a**_**1**_ Operational procedures during the implantation of the sensor in LPS-induced fever model mice. **a**_**2**_ Monitoring of the subcutaneous temperature of mice (blue line), and the corresponding ΔR/R_0_ of the sensor (black line) after intraperitoneal injection of LPS. Reproduced with permission. Copyright 2022, American Chemical Society. **b** Amperometric temperature monitoring [180]. **b**_**1**_, **b**_**2**_ The current variation of PDRG hydrogel-based temperature sensor before and after the “artificial fever”. **b**_**3**_, **b**_**4**_ Recording volunteer's forehead temperature through infrared thermal images of the human forehead before and after the “artificial fever”. Reproduced with permission. Copyright 2012, Royal Society of Chemistry. **c** Thermochromic temperature monitoring [182]. **c**_**1**_ Thermochromic mechanism of thermochromic microcapsules. **c**_**2**_ SEM image of thermochromic microcapsules. Reproduced with permission. Copyright 2020, American Chemical Society

**Resistive Temperature Monitoring. **Pang et al. [179] prepared a dual-network, stretchable and temperature-responsive ionic conductive hydrogel by introducing a polyvinylpyrrolidone (PVP)/tannic acid (TA)/Fe^3+^ cross-linked network into the MBA cross-linked poly(N-isopropylacrylamide-co-acrylamide) (PNA) network. The temperature-conductivity responsiveness of PNA/PVP/TA/Fe^3+^ hydrogel derived from PNIPAAm makes it suitable for wearable temperature monitoring. Interestingly, the PNA/PVP/TA/Fe^3+^ hydrogel possesses temperature-responsive volumetric phase transition properties owing to the introduction of PNIPAAm. When the temperature is lower than VPTT (Volumetric Phase Transition Temperature), the movement of ions inside the hydrogel accelerates with increasing temperature, eventually leading to a decline in resistivity. When the temperature is higher than VPTT, the hydrophobic interaction of PNIPAAm polymer chains is enhanced, resulting in shrinkage and entanglement between PNIPAAm polymer chains, and water molecules are separated from the polymer chains. The higher the temperature, the more water molecules are excluded and the higher the resistance of the hydrogel. Based on this principle, the resistivity of the PNA/PVP/TA/Fe^3+^ hydrogel-based temperature sensor showed a "V-shaped" change with increasing temperature. The VPTT of PNA/PVP/TA/Fe^3+^ hydrogel can be adjusted by changing the amount of AM added during the polymerization of the P(NIPAAm-co-AM) network, thereby adjusting the inflection point of the conductivity-temperature curve. At the 5:1 ratio of NIPAAm: AM, the resistance–temperature curve of the hydrogel has a "V-shaped" inflection point close to the physiological temperature (37 °C), so the hydrogel can be used as a wearable temperature sensor for real-time monitoring body temperature and detecting human fever or infection. The research team induced systemic fever by injecting lipopolysaccharide (LPS) into a mouse and used PNA/PVP/TA/Fe^3+^ hydrogel-based temperature sensors to monitor temperature changes in real-time (Fig. 10a_1_). As can be seen from Fig. 10a_2_, when the temperature exceeds the normal temperature level, there is an inflection point in the impedance curve of the sensor, which can be considered a warning of hyperthermia.

**Amperometric Temperature Monitoring.** Chen et al. [180] prepared organohydrogel materials with high toughness (3.1 MPa @ 600% strain) and high electrical conductivity by adding reduced graphene oxide (rGO) and graphene oxide (GO) nanosheets to PVA hydrogel network within dimethyl sulfoxide (DMSO)/H_2_O binary solvent. The prepared PDRG (PVA/DMSO/RGO/GO) organohydrogel possesses excellent sensitivity and linear repeatable response ability in both mechanical and thermal stimulation. The combination of DMSO and H_2_O molecules can effectively inhibit the crystallization and evaporation of water, so the strain/pressure sensor and temperature sensor prepared using this organohydrogel have a wide working temperature range (− 30 to 60 °C). The organohydrogel temperature sensor was attached to the subject's forehead to detect the body temperature variation, proving the feasibility of hydrogel sensor for body temperature monitoring. The researchers used a calibrated IR camera to record the volunteer's forehead temperature before and after the "artificial fever" and used an organohydrogel temperature sensor to collect the corresponding electrical signals. As can be seen from Fig. 10b_1_–b_4_, when the subject's forehead temperature was 36.2 °C, the corresponding current was 22.9 μA. When the subject’s forehead temperature rose to 39.6 °C, the corresponding current increased rapidly to 25.3 μA. When the forehead temperature dropped to the initial temperature, the current signal can recover well to the initial value. Li et al. [181] have conducted much research to improve hydrogel's antifreeze and moisturizing properties. They studied the antifreeze and moisturizing properties and mechanisms of aqueous solutions of glycerol (Gly), ethylene glycol (EG) and CaCl_2_, and finally chose glycine solution as antifreeze and moisturizing agents. The researchers finally prepared a wearable temperature sensor with high sensitivity, fast response, high precision and good stability by introducing Gly and silver nanofibers (AgNW) into PVA gel.

**Thermochromic Temperature Monitoring.** The composite hydrogel-based temperature sensors described above are all based on the principle of conductivity-temperature response. Some researchers have also tried to use the thermochromic principle for temperature monitoring. For example, Chen et al. [182] reported a core–shell segment-structured multifunctional conductive hydrogel/thermochromic elastomer hybrid fiber for wearable strain and temperature sensing. Reduced-graphene-oxide-doped poly(2-acrylamido-2-methyl-1-propanesulfonic acid-co-acrylamide) (rGO-poly(AMPS-co-AAm)) hydrogel and a thermochromic elastomer containing silicon rubber and thermochromic microcapsules were used as strain-sensitive and thermosensitive materials, respectively. They fabricated conductive hydrogel/thermochromic elastomer hybrid fibers with core–shell segmental configuration via dual-core coaxial wet spinning. According to the trichromatic theory that any color can be composed of three primary colors, thermochromic elastomers were finally obtained by mixing three thermochromic elastomers containing monocomponent blue, red, or green thermochromic microcapsules in a ratio of 4:1:9. Apart from being affected by temperature, the electrical conductivity of hydrogel materials is also disturbed by various external factors such as strain and humidity. The reversible color change of temperature sensors based on the thermochromic principle depends on the formation or destruction of a colored complex between the coloring agent and the developer (Fig. 10c_1_, c_2_) and thus is less affected by environmental factors and has better performance stability than the temperature sensor based on the conductivity-temperature response.

### Wound Healing Monitoring

Wounds are susceptible to bacterial infection during the healing process. Wound infection often leads to slow healing, inflammation, severe suppuration, and even tissue necrosis [183–185]. Traditional wound management usually separates wound healing and monitoring. The wound is first disinfected and treated with drugs, and then the degree of wound healing is assessed during regular dressing changes. This treatment mode will not only cause secondary injury to the patient's wound but may also lead to delayed dressing changes due to failure to monitor wound healing in real time [186–188]. Therefore, how to perform real-time monitoring of wounds while healing remains a major challenge for clinical tissue regeneration. Hydrogel materials with high water content, good biocompatibility, and structure similar to human soft tissue are excellent wound dressings for promoting wound healing [188–190]. After contacting human tissue, it can prevent the infection of microorganisms in vitro and the loss of body fluids [190–192]. Besides, it can form a multifunctional composite hydrogel for monitoring wound healing by introducing specific components. Smart composite hydrogel-based sensors for monitoring wound healing are becoming a new research hotspot.

**Fig. 11:**
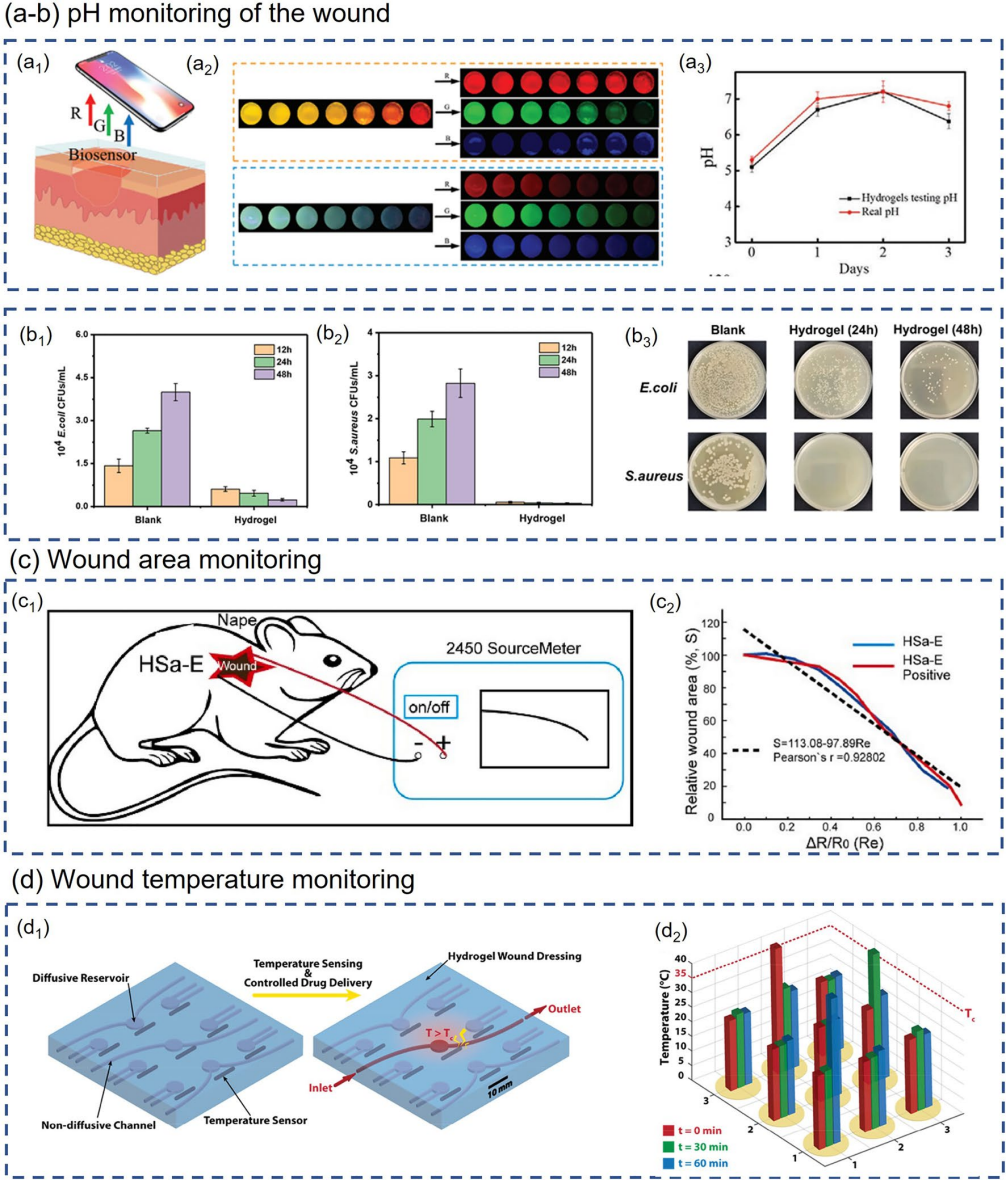
**a**, **b** pH monitoring of the wound [194, 195]. **a**_**1**_ Reading and monitoring the pH value of hydrogels using smartphones. **a**_**2**_ RGB images of PAM-QCS-C-P hydrogels at different pH values under visible light and UV exposure. **a**_**3**_ pH variation of mice wound. Reproduced with permission. Copyright 2021, John Wiley and Sons. **b**_**1**_, **b**_**2**_ Quantitative results of the antibacterial properties of the multifunctional hydrogels against *E. coli* and *S. aureus*. **b**_**3**_ Bacterial colonies of *E. coli* and *S. aureus* after 24 h, 48 h contact with hydrogels, respectively. Reproduced with permission. Copyright 2022, Elsevier. **c** Wound area monitoring [196]. **c**_**1**_ Diagram of the wound healing and monitoring experiment. **c**_**2**_ Correlation curve of relative wound area and resistance changes and in HSa-E group and HSa-E/Positive group. Reproduced with permission. Copyright 2022, Elsevier. **d** Wound temperature monitoring [198]. **d**_**1**_ The temperature sensors are patterned into a 3 × 3 matrix with a drug-delivery reservoir next to each of them. The smart wound dressing can give programmable and sustained deliveries of different drugs at various locations over human skin according to the temperatures measured at those locations. **d**_**2**_ The temperatures at different locations on the skin are measured via wireless temperature sensor over time. Reproduced with permission. Copyright 2015, John Wiley and Sons

**pH Monitoring of the Wound. **In the wound healing process, pH value is a vital monitoring indicator, reflecting the wound infection and the degree of tissue regeneration. Normal skin with a pH of 4–6 is a natural barrier against microbial infection. Conversely, after infection, the pH of the wound increases to 7–8 due to the action of microorganisms and their enzymes [192, 193]. Zheng et al. [194] first physically cross-linked quaternary ammonium chitosan (QCS) and PAM to construct PAM-QCS hydrogels. The CQDs and pH indicator (phenol red) were then loaded into the PAM-QCS gel by the diffusion hybridization method. Inside the prepared PAM-QCS-C-P hydrogels, PAM and QCS endow the hydrogel with excellent biocompatibility and antibacterial properties, respectively. The two form a cross-linked network through physical entanglement. pH-sensitive CQDs and phenol red were used to construct a two-color monitoring system based on visible color and fluorescent signals. As shown in Fig. 11a_1_, Zheng et al. used a smartphone to collect pictures of PAM-QCS-C-P hydrogels and converted them into RGB signals for remote processing, significantly improving the accuracy of pH monitoring. It can be seen from Fig. 11a_2_ that under visible light, as the pH value increases, the R value of the PAM-QCS-C-P hydrogel increases, the G value decreases, and the B value remains unchanged. G/B is used as the signal corresponding to the pH value. By plotting the relationship between G/B and pH value, the corresponding Eq. (5) is obtained by fitting. When under UV light, the RGB value of the hydrogel will decrease with the increase of pH, and the final fit yields Eq. (6). The researchers applied the hydrogel to the wound to further study its ability to monitor wound healing. It can be seen from Fig. 11a_3_ that the pH value measured by the hydrogel dual colorimetric detection system is consistent with the change in the actual value.

5 $$\begin{array}{*{20}c} {{\text{pH}} = 29.21 - 6.76\sqrt { - 2\ln \frac{{3.4 - \frac{G}{B}}}{430.92}} } \\ \end{array}$$

6 $$\begin{array}{*{20}c} {{\text{pH}} = 8.65 - \frac{G + B}{{94.64}}} \\ \end{array}$$

Wang et al. [195] also designed a pH-responsive HACC-PAM hydrogel for intelligent wound monitoring. The intelligent wound detection system constructed by the research team is divided into wound recognition, real-time status monitoring and personalized wound management. First, hydrogel wound dressings that can accurately match irregularly-shaped wounds were prepared through online wound scanning and offline smart printing. Next, litmus reagent was introduced into the hydrogel by solvent displacement, enabling wound pH detection by colorimetry. Finally, the chrominance signal was converted into a pH-sensing image using a smartphone and achieved personalized wound management via a CNN (Convolutional Neural Network) machine learning algorithm. Throughout the process, Wang et al. focused on the antibacterial, hemostasis, and wound-healing abilities of HACC-PAM hydrogels. Common pathogenic microorganisms in skin wounds, such as *E. coli* (Gram-negative bacteria) and *S. aureus* (Gram-positive bacteria), were used to verify the antibacterial properties of hydrogels. It can be seen from Fig. 11b_1_–b_3_ that the number of colony-forming units (CFU) of Escherichia coli and Staphylococcus aureus in the petri dishes were significantly reduced after co-culturing with the multifunctional hydrogel for 12, 24, and 48 h. The research team also used a mouse hemorrhaging liver model to evaluate the hemostatic ability of multifunctional hydrogel. The experimental results showed that the total blood loss in the multifunctional hydrogel group was much lower than that in the control group (53.8 mg) within 60 s. In addition, compared with the wounds of mice infected with Staphylococcus aureus without any treatment and covered with multifunctional hydrogel, the wound healing speed and regeneration quality of the latter were better than those of the former, which demonstrated the advantage of HACC-PAM in promoting wound regeneration.

**Wound Area Monitoring. **The wound area usually represents the degree of healing. Some researchers try to monitor wound healing by detecting the wound area. For example, Chen et al. [196] prepared HSa hydrogels in simple steps using chitosan quaternary ammonium salt (HACC) and sodium alginate (SA) as raw materials. HACC and SA form a "Magic Cube" like structure through the electrostatic interaction between the positive charge of the amino group in HACC and the negative charge of the carboxylic acid group in SA, which can arbitrarily transform like a Rubik's cube. Due to free Cl^−^, the HSa hydrogel has good electrical conductivity (1.14 × 10^−3^ S cm^−1^), and its resistance change has a positive linear relationship with the area change. Because of the features mentioned above, the HACC-SA (HSa) hydrogel can be used to measure the wound area to assess wound healing. The researchers applied the hydrogel to the mouse's wound and used a digital meter to measure the resistance change of the hydrogel (Fig. 11c_1_). Figure 11c_2_ shows that the relative wound area change has a positive linear correlation with the recorded relative resistance change ΔR/R_0_, which proves the feasibility of HSa hydrogel for wound detection. In addition, Chen et al. also investigated the ability of HSa hydrogels to promote wound healing. The wound healing performance of the HSa group was better than that of the control group on Day 6, Day 9 and Day 12, demonstrating that HSa hydrogel can effectively promote wound healing in vivo.

#### Wound Temperature Monitoring

**Wound Temperature Monitoring.** Wound temperature is important parameter for wound detection. An elevated temperature means the wound may be infected, inflamed, or engorged. Wound temperature decrease may indicate low collagen deposition, and reduced late-phase regenerative inflammatory cells and fibroblasts [197]. Lin et al. [198] designed a smart wound dressing with temperature sensors, drug delivery channels and reservoirs for wound healing monitoring and drug delivery. The smart dressing was obtained through patterning temperature sensors, non-diffusive drug-delivery channels (made of plastic tubes), and diffusive drug reservoirs in the hydrogel matrix. Its device structure and working principle are shown in Fig. 11d. The as-prepared intelligent dressing simultaneously has the functions of wound detection and drug release control. When the sensor detects that the temperature at a certain location rises above a threshold (such as 35 °C), the drug solution can be manually delivered to the corresponding drug reservoir through the non-diffusion drug delivery channel and then diffuse out of the hydrogel matrix in a controlled and sustained manner. This study provides a new idea for the design of composite hydrogel electronics and devices.

### Disease Diagnosis

As people pay more and more attention to health, the public has also put forward more personalized needs for wearable medical devices. In addition to monitoring the user's physical condition in real time, auxiliary disease diagnosis is also a crucial part of wearable health monitoring. The wearable health monitoring system can collect the user's relevant information and process it locally or transmit it to the remote medical service center. It provides a simple and convenient self-diagnosis method for the patient and can assist the physician in rapid disease diagnosis.

#### Cancer

**Fig. 12:**
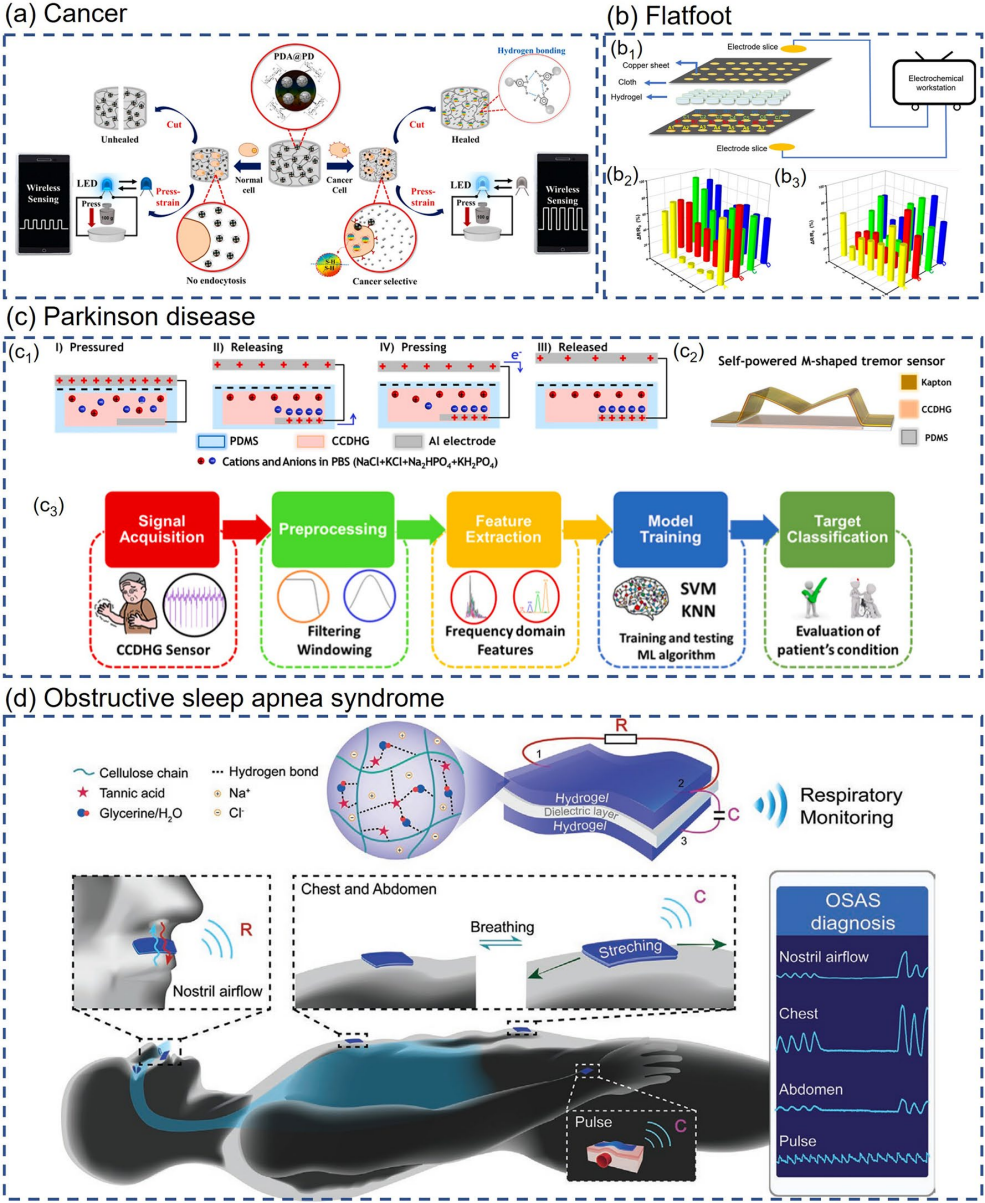
**a** Cancer [199]. Schematic illustration of selective strain-pressure and cancer-mediated self-healing phenomenon of hydrogel-based sensor for cancer detection with real-time monitoring via smartphone. Reproduced with permission. Copyright 2021, Elsevier. **b** Flatfoot [162]. **b**_**1**_ Schematic diagram of the hydrogel pressure sensor insole. The insole was divided into 28 areas, numbered A1–A7, B1–B7, C1–C7, and D1–D7, and copper sheets and hydrogels were placed in these areas to monitoring plantar pressure distribution. **b**_**2**_, **b**_**3**_ Three-dimensional histograms of the relative resistance of hydrogel compressive sensor for monitoring the normal foot and flatfoot. Reproduced with permission. Copyright 2015, Elsevier. **c** Parkinson disease [201]. **c**_**1**_ Working mechanism of CCDHG-TENG. **c**_**2**_ Schematic image of self-powered M-shaped tremor sensor. **c**_**3**_ Flow chart for evaluating condition of PD patient. Reproduced with permission. Copyright 2021, Elsevier. **d** Obstructive sleep apnea syndrome [202]. Schematic illustration of cellulose based hydrogel sensor with multimodal sensing capability for monitoring respiration. Reproduced with permission. Copyright 2022, John Wiley and Sons

Robby et al. [199] proposed a cancer cell-selective wireless strain and pressure sensor. First, they prepared a polymer dot (PD) by hydrothermal carbonization of disulfide-crosslinked hyaluronic acid (HA-S-S-HA). Then polydopamine (PDA) was added to PD through hydrophobic interaction to obtain hybrid PDA@PD nanoparticles. Finally, PDA@PD-PAM hydrogel was prepared by incorporating hybrid PDA@PD nanoparticles into the PAM hydrogel. Interestingly, PDA@PD-PAM hydrogel exhibits selective strain-pressure response and self-healing phenomena in the presence of cancer cells, which make it feasible to monitor cancer cells (Fig. 12a). The detection principle is as follows: Excessive glutathione (GSH) in cancer cells leads to disulfide bond cleavage in PDA@PD and release of PDA. The released PDA would form hydrogen-bonding interactions inside the hydrogel to promote the self-healing of the hydrogel. In addition, PDA also induces changes in the electrical conductivity and strain-pressure response of PDA@PD-PAM hydrogel. By connecting the PDA@PD-PAM hydrogel and wireless electronic devices, the real-time data of self-healing and pressure sensing can be directly recorded through a smartphone to distinguish normal cells and cancer cells. Robby et al. [200] have conducted more research on the application of cancer cell detection based on hydrogels. Unlike the previous GSH-responsive self-healable conductive hydrogel, the research team designed a pH-triggered controllable conductive hydrogel for cancer cell detection. CD-PNB@PVA hydrogel exhibits different conductivities and strain-pressure responses under acidic conditions, owing to the cleavage of diol-diol cross-linking between the catechol in semiconducting CD (carbon dots) and the boronic acid group in PNB (Pluronic-grafted-poly(DMA-co-NIPAAM)-boronic acid). Since the microenvironment of cancer cells is more acidic (pH < 6.8) than normal cells (pH $\approx$ 7.4), hydrogels attached to cancer cells have higher electrical conductivity and strain-pressure response than hydrogels attached to normal cells, which is an essential criterion for distinguishing cancer cells from normal cells.

#### Flatfoot

Flatfoot is a foot deformity characterized by a low or disappearing foot arch. Due to the lack of cushioning in the foot arch, patients with flat feet are prone to discomfort when walking for a long time. Patients with flat feet have different plantar pressure distributions from ordinary people. A plantar pressure sensor made of the compressive strain-sensitive hydrogel can monitor the user's plantar pressure distribution to identify flat feet. Ling et al. [162] prepared a multifunctional conductive double network hydrogel pressure sensor that can be exploited in the diagnosis of flatfoot. The DCMC/AG/PAA DN hydrogel has excellent compressive properties (the strength reached to 0.12 MPa when the hydrogel was compressed to 50% of its original height), reliable electrical conductivity, and high sensitivity (Gauge Factor = 8.1), making it suitable for plantar pressure monitoring. The researchers cut out 28 areas on the cloth, numbered A1–A7, B1–B7, C1–C7, and D1–D7, and placed copper sheets and hydrogels in these areas. Finally, the electrode sheet of the electrochemical workstation was connected to the copper sheet to record the signal generated by the plantar pressure (Fig. 12b_1_). Comparing the plantar pressure distribution of flat foot patients and healthy people, the skeleton of the normal foot is arched, and the pressure at the A2, A3, A4, and A5 positions is the lowest, so the resistance value is almost unchanged. On the other hand, flat feet are subject to tremendous pressure in areas A2, A2, A3, A4, and A5, and these areas have higher resistance values (Fig. 12b_2_, b_3_).

#### Parkinson Disease

Kim et al. [201] first synthesized a stretchable and self-healable hydrogel conductor using catechol, chitosan, and diatoms as raw materials, which can be exploited as a triboelectric nanogenerator to obtain energy from human motion. The catechol-chitosan-diatom hydrogel triboelectric nanogenerators (CCDHG-TENG) were combined with M-shaped Kapton film to construct a self-powered tremor sensor. CCDHG was wrapped by triboelectrically negative PDMS film and then contacted with Al film. Due to the difference in work function, the Al film and the CCDHG surface are positively and negatively charged, respectively. When the two surfaces are separated, the electrostatic charges on the PDMS repel the negative ions and attract the positive ions inside the CCDHG to balance the electrostatic charges on the surfaces. At the same time, electrons attached to the surface of the Al electrode are transferred to the Al film. The process is reversed when the counter material is reattached to the PDMS. Repeated contact and separation between the CCDHG-TENG and the counter material generates alternating current to power the sensor (Fig. 12c_1_). The tremor sensor, composed of an M-shaped Kapton counter layer and CCDHG-TENG, could react sensitively to low-frequency vibrational motions (Fig. 12c_2_). The research team installed the tremor sensor on the wrist of a Parkinson's patient and recorded the voltage signal generated by the tremor. After the voltage–time signal is filtered, the power spectral density (PSD) is used for frequency domain conversion. Then, the corresponding features are extracted from the frequency domain data for subsequent model training, and the patient's condition is classified and evaluated through algorithms such as KNN (K-Nearest Neighbors) and SVM (Support Vector Machines) (Fig. 12c_3_). It can be seen that the combination of wearable sensors and artificial intelligence algorithms has dramatically improved the accuracy of disease diagnosis, which is of great significance for building a powerful wearable health monitoring system.

#### Obstructive Sleep Apnea Syndrome

Breathing usually reflects the health status of the human body, especially closely related to sleep quality and sleep diseases. The traditional respiratory monitoring device is bulky and has low wearing comfort, seriously affecting the wearer's sleep quality. We have introduced several respiration monitoring applications based on smart composite hydrogel sensors in the physiological state monitoring section. These smart composite hydrogel sensors, with good flexibility and stretchability, can fit well with the human body and are very suitable for building wearable respiratory monitoring systems. Liu et al. [202] sandwiched a commercial elastomer (VHB 4905, 3 M) between two CH-GT hydrogel layers as a dielectric film to form a multimodal hydrogel sensor (Fig. 12d). When electrodes 2 and 3 are connected, a parallel-plate capacitive sensor sensitive to compression or stretching is generated; when electrodes 1 and 2 are connected, due to the temperature-sensitive ion dissociation and transport behavior inside the hydrogel, a temperature-sensitive ionic resistance sensor is formed. Obstructive sleep apnea syndrome (OSAS) is a sleep disorder in which breathing stops intermittently during sleep due to pharyngeal narrowing. In order to monitor OSAS, Liu et al. simultaneously attached four CH-GT100 hydrogel sensors to corresponding body parts to independently monitor nostril airflow, chest and abdomen movement, and pulse. When an apnea occurred, sensors attached to the upper lip, chest, and abdomen recorded an almost flat signal, indicating an obstructive respiratory event. Liu et al. applied multimodal hydrogel to multiple body parts for simultaneous monitoring, eliminating the interference of a single electrical signal or a single body part factor, significantly improving the accuracy of respiratory monitoring and the reliability of respiratory disease diagnosis.

**Table 3:** Summary of sensing mechanisms for health monitoring

Application	Composite hydrogel	Target	Sensing mechanism
Sweat monitoring	BC/CMC hydrogel [143]	pH, glucose	Colorimetry
	M-hydrogel [148]	pH	Resistance
	TOCNF/PANI-PVA hydrogel [149]	Ions	Triboelectricity
	PVA-ATMP hydrogel [150]	Ions	Hofmeister effect
Respiratory monitoring	PAM/CS hydrogel [203]	Strain	Resistance
	PAM/alginate hydrogel [161]	Pressure	Capacitance
	DCMC/AG/PAA hydrogel [162]	Strain	Resistance
	PDA–clay–PSBMA hydrogel [163]	Strain	Resistance
	starch/PAM hydrogel [164]	Humidity	Conductance
	PAM/CA hydrogel [26, 165]	Humidity	Capacitance
	PVA/GEL hydrogel [166]	Temperature	Thermoelectricity
Pulse monitoring	PANI/P(AAm-co-HEMA) hydrogel [100]	Strain	Resistance
	PVA-HEDP hydrogel [172]	Strain	Resistance
Heartbeat monitoring	Alg-CNT hydrogel [173]	Strain	Amperometry
	OPPC hydrogel [119]	Strain	Resistance
Body temperature monitoring	PNA/PVP/TA/Fe^3+^ hydrogel [179]	Temperature	Resistance
	PVA/DMSO/RGO/GO hydrogel [180]	Temperature	Amperometry
	rGO-poly(AMPS-co-AAm) [182]	Temperature	Colorimetry
Wound healing monitoring	PAM-QCS-C-P hydrogel [194]	pH	Colorimetry
	HACC-PAM hydrogel [195]	pH	Colorimetry
	HSa hydrogel [196]	Area	Resistance
Cancer	PDA@PD-PAM hydrogel [199]	glutathione	Conductance
Cancer	CD-PNB@PVA hydrogel [200]	pH	Conductance
Flatfoot	DCMC/AG/PA hydrogel [162]	Pressure	Resistance
Parkinson disease	Catechol-chitosan-diatom hydrogel [201]	Tremor	Electric potential
Obstructive Sleep Apnea Syndrome	CH-GT hydrogel [202]	Pressure, strain, temperature	Capacitance, Resistance

## Conclusions and Outlook

By adjusting the internal components of hydrogels or constructing unique hydrogel structures through hydrogel engineering, smart composite hydrogels with a variety of properties, such as enhanced mechanical properties, electrical conductivity, self-healing, stimuli responsiveness, and self-adhesion, can be obtained. Smart composite hydrogels, which possess excellent physicochemical properties, have great potential in wearable sensing fields [204, 205]. Wearable health monitoring devices based on smart composite hydrogels have been widely used in health care scenarios such as physiological state monitoring, wound monitoring, and disease diagnosis. Among them, physiological state monitoring mainly focuses on the user's physiological activities. It aims to evaluate the user's health status by recording various physiological indicators, which are significant for health management, disease prevention, and disease control. Furthermore, due to the excellent biocompatibility, antibacterial properties and ability to promote wound healing, smart dressings prepared with composite hydrogels can not only monitor wound healing in real time but also prevent infection and promote wound healing. In addition, disease diagnosis is also an essential part of wearable health monitoring. Smart composite hydrogel-based wearable sensors have been proven to monitor and diagnose diseases such as cancer, flat feet, Parkinson's, and obstructive sleep apnea syndrome. Smart composite hydrogel, as one of the most potential flexible electronic materials, is expanding the limits of what is possible.

Although smart composite hydrogel-based sensors have many advantages, there are still deficiencies at this stage. These defects limit the practical application of composite hydrogels in wearable health monitoring and even become an obstacle in the industrialization of composite hydrogel-based sensors. In wearable health monitoring applications, we often pay attention to wearable sensors' long-term performance stability, i.e., operating continuously for a long time in various complex environments without apparent performance degradation. The evaporation and condensation of water and the corrosion of metal electrodes are essential factors affecting the long-term performance stability of hydrogel-based sensors [206, 207].

Among them, improving the antifreeze and moisture retention properties of composite hydrogels is one of the problems to be solved urgently. Traditional hydrogel materials contain a large amount of water. This free water will evaporate in a high-temperature dry environment and condense in a low-temperature environment. The instability of the solvent inside the hydrogel material will not only significantly impair the mechanical properties of the sensor but also affect the performance stability of the sensor. Therefore, the application scenarios of composite hydrogel-based sensors are usually harsh. The current mainstream solution is to replace the internal water with glycerol (Gly), ethylene glycol (EG) or CaCl_2_ aqueous solution through a solvent replacement strategy. Glycerol and ethylene glycol molecules contain many hydroxyl groups, which can combine with water and effectively prevent the evaporation and condensation of water. Unlike Gly and EG, the CaCl_2_ aqueous solution fixes water firmly in the polymer network through the strong coordination bond between water and calcium (II). However, the effects of these methods are minimal, especially the problem of slow water loss during long-term use of the sensor is hard to solve. Further research efforts should be devoted to improving the stability of hydrogel and related sensing devices.

In addition to the issue of antifreeze and moisture retention, electrode corrosion affects sensor performance as well. Due to the long-term application of voltage, especially DC voltage on the electrodes, the surface of the sensor electrodes is prone to corrosion and the accumulation of reaction products, which seriously decrease the performance stability and service life of the sensor. Especially when composite hydrogel-based sensors are used to monitor biomarkers such as secretions in sweat or enzymes and receptors in tissues and organs, the electrodes are often severely corroded due to electrochemical side reactions. The performance degradation of the sensor caused by electrode corrosion makes it difficult for the smart composite hydrogel-based sensor to adapt to the long-term uninterrupted operation requirements of the wearable health monitoring system. It is expected to develop effective strategy to improve the long-term stability of electrode and hydrogel-electrode interface. In the composite hydrogel-based sensor, the metal electrode persistently suffered from water-induced corrosion reactions due to the direct contact with the hydrogel. Some researchers tried to partially replace the water in the hydrogel using a solvent replacement strategy to reduce the free water content and water activity, thereby prolonging the electrode life [208]. Furthermore, introducing a hydrophobic film on the surface of the metal electrode to isolate the electrode from the aqueous environment is  a common strategy to solve electrode corrosion [209]. In addition to water-induced corrosion, adsorption and side reactions of free ions on the electrode can also lead to severe corrosion [210]. Therefore, using ion-free composite hydrogel as the electrolyte of the sensor can effectively prolong the service life of the electrode. For example, Tang et al. [211] attempted to introduce charged GO/rGO in composite hydrogels as the electrolyte. GO/rGO forms a stable network structure in the hydrogel through hydrogen bonding and physical entanglement, so it will not move toward the electrode under the action of an electric field, reducing the corrosion of the electrode.

Furthermore, insufficient air permeability is also a significant problem for hydrogel-based sensors. Although the composite hydrogel can be biocompatible after being regulated by hydrogel engineering, its long-term adhesion to the skin surface will prevent "skin respiration", resulting in inflammation. Developing hydrogel film with ultralow thickness or porous hydrogel may be a solution to this issue [212–214]. When a hydrogel covers the skin, the gas exchange between the skin and the external environment must rely on the diffusion of the hydrogel interface. The short diffusion distance of the ultrathin hydrogel film or the porous structure inside the hydrogel dramatically shortens the time for water vapor and gas to go across the interface, thereby realizing a wearable hydrogel-based sensor with good air permeability.

When composite hydrogel sensors are attached to the body surface, extra strain signals are often generated due to collisions, scratches, or inadvertent actions of the wearer, which in turn interferes with the response signals of the sensors. Preparing strain-insensitive sensors, or decoupling the valid strain signals of sensors from interference signals, is the key to constructing a wearable health monitoring system with high precision and anti-interference. Furthermore, the multi-stimuli responsiveness of composite hydrogels often leads to unwanted noise or coupling between multiple stimuli. Data processing methods based on machine learning (ML) can efficiently process a large amount of data generated by sensors and discover intricate and hidden relationships in the data [215, 216]. The ML model trained by feeding a large amount of data can effectively reduce noise or decouple multiple stimulus signals.

Furthermore, most of the previously reported smart composite hydrogel-based sensors are large. Due to the lack of high-precision and standardized processing technology, the performance consistency of fabricated sensors cannot be guaranteed and it is challenging to produce in large quantities. Therefore, developing highly accurate and standardized preparation methods is significant for commercializing smart composite hydrogel-based sensors. The standardized preparation of composite hydrogel sensors ensures the reliability and consistency of the device's performance. The miniaturization of the device means smaller volume, lower weight, and lower power consumption of the sensor. Moreover, integrated technique can integrate different sensors or actuators to form a microsensor array or microactuator array, thereby constructing a wearable medical microsystem with multiple functional modules such as real-time monitoring, data transmission, timely drug delivery and treatment. One method to realize mass fabrication, miniaturization and integration of hydrogel-based sensors is to combine photocurable hydrogel with photolithography technology to prepare micropatterned hydrogel through specific mask exposure [217–219]. In addition, microfluidics [220, 221], 3D printing [221, 222], and non-contact forces [223, 224] (i.e., electric, magnetic, or acoustic fields and self-assembly) are also competitive to be exploited in fabricating hydrogel-based devices with complex structures.

Although there are still many limitations in applying smart composite hydrogel-based sensors, the problems mentioned above will likely be solved soon with the continuous exploration of research teams worldwide. More application scenarios for smart composite hydrogel-based sensors will be unlocked at that time (Tables 1, 2 and 3).
